# Travel time to health facilities in areas of outbreak potential: maps for guiding local preparedness and response

**DOI:** 10.1186/s12916-019-1459-6

**Published:** 2019-12-30

**Authors:** E. N. Hulland, K. E. Wiens, S. Shirude, J. D. Morgan, A. Bertozzi-Villa, T. H. Farag, N. Fullman, M. U. G. Kraemer, M. K. Miller-Petrie, V. Gupta, R. C. Reiner, P. Rabinowitz, J. N. Wasserheit, B. P. Bell, S. I. Hay, D. J. Weiss, D. M. Pigott

**Affiliations:** 1 Institute for Health Metrics and Evaluation, University of Washington, 2301 5th Ave, Suite 600, Seattle, WA 98121 USA; 2 Malaria Atlas Project, Big Data Institute, Li Ka Shing Centre for Health Information and Discovery, Oxford, UK; 3 Institute for Disease Modeling, Bellevue, WA 98005 USA; 4 Department of Zoology, University of Oxford, Oxford, UK; 5 Department of Health Metrics Sciences, School of Medicine, University of Washington, Seattle, WA 98121 USA; 6 Department of Global Health, University of Washington, Seattle, WA 98195 USA

**Keywords:** Preparedness, Viral hemorrhagic fevers, Travel time, Health facility access, Ebola, Marburg, Lassa fever, CCHF

## Abstract

**Background:**

Repeated outbreaks of emerging pathogens underscore the need for preparedness plans to prevent, detect, and respond. As countries develop and improve National Action Plans for Health Security, addressing subnational variation in preparedness is increasingly important. One facet of preparedness and mitigating disease transmission is health facility accessibility, linking infected persons with health systems and vice versa. Where potential patients can access care, local facilities must ensure they can appropriately diagnose, treat, and contain disease spread to prevent secondary transmission; where patients cannot readily access facilities, alternate plans must be developed. Here, we use travel time to link facilities and populations at risk of viral hemorrhagic fevers (VHFs) and identify spatial variation in these respective preparedness demands.

**Methods and findings:**

We used geospatial resources of travel friction, pathogen environmental suitability, and health facilities to determine facility accessibility of any at-risk location within a country. We considered in-country and cross-border movements of exposed populations and highlighted vulnerable populations where current facilities are inaccessible and new infrastructure would reduce travel times. We developed profiles for 43 African countries. Resulting maps demonstrate gaps in health facility accessibility and highlight facilities closest to areas at risk for VHF spillover. For instance, in the Central African Republic, we identified travel times of over 24 h to access a health facility. Some countries had more uniformly short travel times, such as Nigeria, although regional disparities exist. For some populations, including many in Botswana, access to areas at risk for VHF nationally was low but proximity to suitable spillover areas in bordering countries was high. Additional analyses provide insights for considering future resource allocation. We provide a contemporary use case for these analyses for the ongoing Ebola outbreak.

**Conclusions:**

These maps demonstrate the use of geospatial analytics for subnational preparedness, identifying facilities close to at-risk populations for prioritizing readiness to detect, treat, and respond to cases and highlighting where gaps in health facility accessibility exist. We identified cross-border threats for VHF exposure and demonstrate an opportunity to improve preparedness activities through the use of precision public health methods and data-driven insights for resource allocation as part of a country’s preparedness plans.

## Introduction

The West Africa Ebola outbreak of 2014–2016, unprecedented among Ebola outbreaks in its morbidity, mortality, and magnitude, was a turning point in epidemic preparedness and global health security [1, 2]. Since the outbreak, several efforts have been launched or reinvigorated to advance preparedness and improve national capacities to prevent, detect, and respond to infectious disease threats [3, 4]. Building on the International Health Regulations [5], the Global Health Security Agenda, in collaboration with the World Health Organization (WHO), introduced the Joint External Evaluations (JEEs) as one mechanism to evaluate current capabilities [6]. This process establishes a checklist for change by evaluating national-level preparedness and vulnerabilities, highlighting and prioritizing important next steps for each country [7, 8]. The JEEs also task national governments with creating or revising “National Action Plans” to define and implement strategies to address gaps and strengthen relevant capacity [7, 8].

National-level evaluations can mask subnational variation in preparedness, however. In scenarios where local outbreaks have resulted in widespread epidemics, local vulnerabilities—including limited capacity to detect or respond to an outbreak—may have significant national and global health security ramifications. While the JEEs—and many National Action Plans—provide a strong foundation for advancing preparedness at the national level, they lack the ability to capture subnational vulnerabilities systematically.

Globally, the ability to prevent, detect, and respond to diseases of pandemic potential requires access to timely and high-quality healthcare from resilient health systems [2, 9, 10]. Therefore, we can consider preparedness requirements as twofold: (1) physical access, or whether populations at risk for pathogen exposure can reach a health facility, and (2) capacity, whether a health facility can effectively diagnose, treat, and prevent further disease transmission from such populations presenting for care. Inadequate access to readily available healthcare has been shown to influence a number of health activities and outcomes, including lower rates of follow-up [11], increased infectious disease morbidity [12], higher mortality [13, 14], and lower vaccination rates, allowing for the potential for increased local transmission [15]. Physical access to health facilities is highly heterogeneous, even within a single country, with undue barriers to accessing care among those living in poor, rural regions [12, 16, 17]. Inadequate transportation and large distances to health facilities, which are generally concentrated in cities or towns [18, 19], are major barriers to obtaining high-quality healthcare [13, 20]. Quality of healthcare and facility resilience, in contrast to geographic accessibility, are a function of the infrastructure, training, and workforce in place in a given facility. Poor healthcare quality has been shown to result in worsened health outcomes, leading to lower survival and premature mortality due to insufficient clinical treatments for patients presenting for care, limited or improper case diagnoses, allowing for the potential for sustained transmission, and suboptimal disease management, among other shortcomings [21, 22]. Large-scale studies have investigated health quality and resilience at a national level [22] but do not provide details at the facility level, while other assessments have targeted facility-specific capacities but are inconsistently conducted and not openly available [23], resulting in an absence of complete facility capacity and quality information for routine use by health planners.

When considering infectious pathogens with high case-fatality rates such as viral hemorrhagic fevers (VHFs), including Ebola, Lassa Fever, Marburg, and Crimean Congo Hemorrhagic Fever (CCHF), rapid diagnosis and timely, high-quality delivery of treatment are critical. This demands not only accessibility to health facilities but also capable staff, high standards of laboratory testing to identify the causal agents, adequate treatment supplies, and appropriate implementation of infection prevention and control (IPC) and other necessary countermeasures to prevent secondary transmission [24, 25]. While systems such as Uganda’s National Viral Hemorrhagic Fever Surveillance and Laboratory Programme have decreased detection lags once a suspected case presents [26], individuals must still engage with a health system to be captured in current reporting mechanisms. Consequently, the ability to differentiate VHFs from other diseases and physical access to a health facility for diagnosis both represent essential dimensions of disease detection. Of equal importance, however, is ensuring that facilities that are most likely to see inpatients with possibly highly contagious pathogens are appropriately prepared. Plans like the WHO Ebola Checklist [27] provide guidance for facilities to ensure adequate equipment is in place to treat VHF cases, as well as training protocols for healthcare workers on how to manage patients appropriately and mitigate their own exposure, and laboratory standards for rapid and accurate detection. Therefore, countries creating or updating preparedness plans need to recognize locations where these two demands exist—(1) where facilities must be capable of detection, treatment, and response, and (2) where vulnerable populations have limited access to existing infrastructure.

**Fig. 1:**
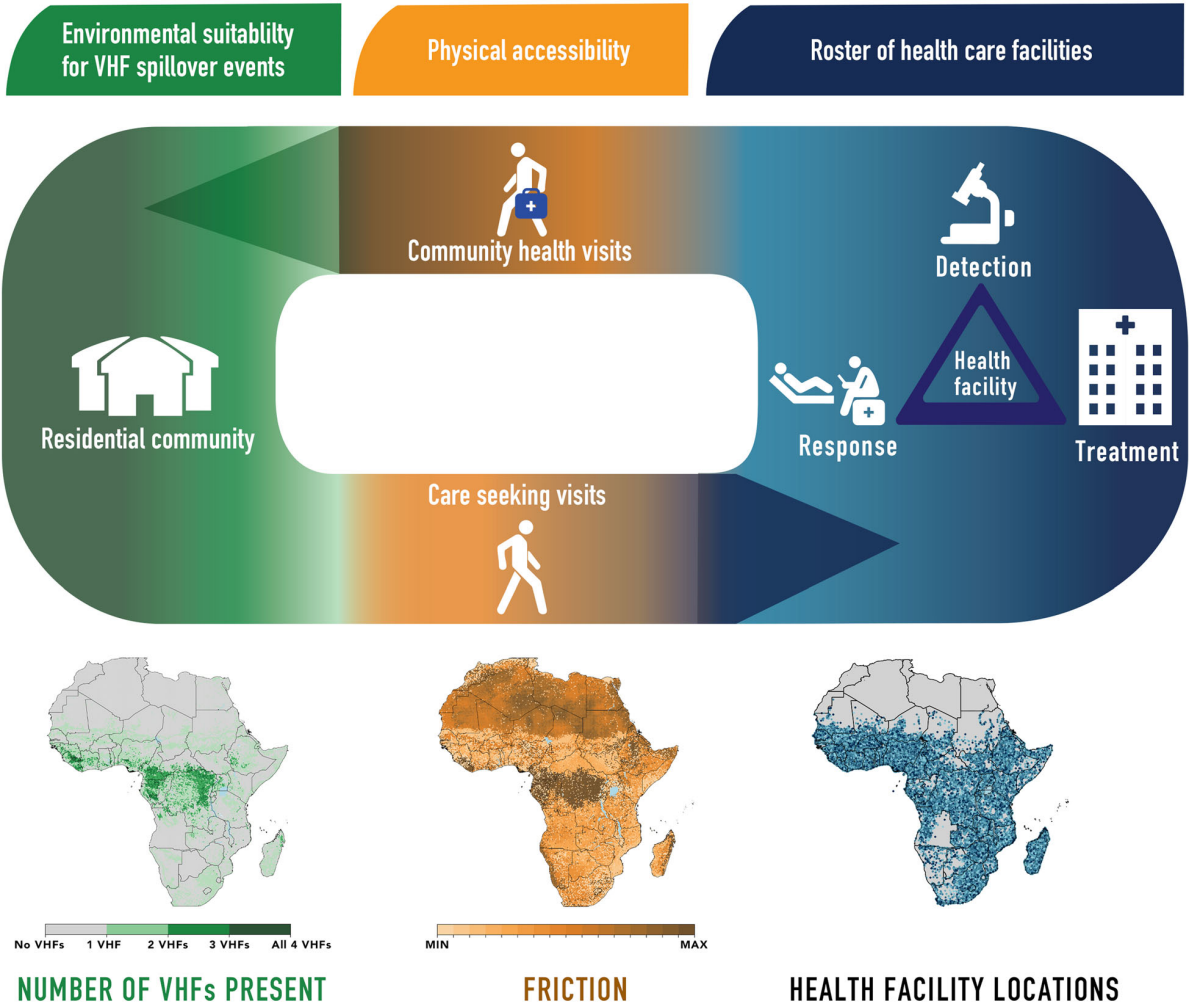
Path from VHF environmental suitability through physical access to health facilities. The key activities from environmental suitability for VHF spillover events, physical accessibility to a health facility, and related detection, treatment, and response are portrayed. At the first stage—environmental suitability, possibilities of spillover events for one or more VHFs are mapped across Africa and demonstrate an individual’s potential for becoming infected with a VHF. At the second stage—physical accessibility, maps quantify how challenging a location’s terrain is to navigate by looking at the topography and relative friction of a surface. At the third stage—heath facility, a roster of health facilities demonstrates the capacities of a health facility (or lack thereof) to detect, treat, and respond to VHF cases. The progression includes a return arrow, as linkage to health facilities can initiate detection, response, and treatment in the community

To identify locations where these distinct demands exist, we can use travel times to quantify populations’ physical accessibility to healthcare infrastructure and identify a priority list of health facilities in close proximity to populations at risk for enhanced capacity. In Fig. 1, we demonstrate this relationship, linking accessibility to healthcare for persons living in locations susceptible to zoonotic transmission. An important feature of this mechanism is that health system accessibility is not merely a function of traveling to hospitals, clinics, and health posts, but is also a function of community response to infectious disease threats.

Recent analyses of VHF spillover event potential (i.e., locations where an index case could arise due to zoonotic transmission) across Africa have demonstrated marked heterogeneities in index case transmission potential and local community susceptibility, both between countries and within a single country [28]. These noted heterogeneities, both national and international, contribute to potential misalignment between locations where outbreaks could originate and health infrastructure—both permanent, such as facilities, and non-permanent, such as highly trained staff or protective equipment; travel time analyses provide insights to this misalignment. Evidence from the past few decades suggests that our increasing connectedness due to international travel and commerce spreads infectious diseases with increasing ease and rapidity [29]. Many borders are porous, and cross-border migration exacerbates transmission risks, allowing those exposed to a disease to travel to locations with no history of zoonotic transmission, as seen with the multi-country spread of the West African Ebola outbreak [30]. Fine-scale geographic preparedness plans for known outbreak-prone pathogens that articulate cross-border risks, in conjunction with facility accessibility maps and known facility capacities, are valuable contributions for local governance and for risk assessments targeting the containment and mitigation of infectious disease outbreaks.

In this study, we build upon existing geospatial assessments of accessibility, contextualizing documented variation in travel times within environmental suitability maps for transmission of outbreak-prone pathogens and locations of health facilities (Fig. 1). Our first aim is to provide a precision public health perspective on where existing facilities are highly accessible from areas potentially at risk or where gaps exist in current health facility accessibility. To assess physical accessibility to health facilities, we quantify the travel times from areas predicted to be at risk of spillover events to the nearest health facility, stratified by type. After identifying populations that may not be served by existing health facilities and those facilities more likely to require improved capacity, we provide spatial foundations for future action, identifying where investing in additional resources—such as new facilities or targeted programs for enhancing surveillance—could have the greatest impact in addressing such gaps as well as a guide for ongoing re-evaluation as improvements are made. Next, as an assessment of proximity of populations to areas with spillover event potential, we quantify travel time from any location in a country to the nearest at-risk area for any VHF. Additionally, given a history of cross-border transmission in previous outbreaks, we look at cross-border mobility of populations, quantifying the travel time from any foreign at-risk location to in-country locations. Finally, noting the gaps in contemporary facility coverage, we identify locations where new infrastructure could have the largest reductions in average travel times for populations living in areas at risk for VHFs. We demonstrate this applicability by using existing environmental suitability maps for four VHFs: Ebola, Lassa, Marburg, and Crimean Congo Hemorrhagic Fever. While the current analyses focus on VHFs in sub-Saharan Africa, these analyses could be expanded to provide data-driven insights into preparedness for any country and any infectious disease so long as a geotagged dataset of health facilities is available.

## Methods

### Data sources

For our analyses, we used four publicly available geospatial sources: (1) environmental suitability rasters (gridded representations of the world) for each of the four VHFs [31–34], (2) a database of geotagged health facilities across the African continent [35], (3) a raster of population by grid-cell [36], and (4) a gridded global friction surface to quantify travel time to facilities [37]. This study complied with the Guidelines for Accurate and Transparent Health Estimates Reporting (GATHER; Additional file 1: Table S1) [38].

### Viral hemorrhagic fever estimates

We used published environmental suitability maps of four VHFs (CCHF [31], Ebola virus disease [32], Lassa fever [33], and Marburg virus disease [34]) to define geographical variation in spillover potential (Fig. 1). These maps utilize geotagged records of viral detections in human and animal populations with gridded covariate datasets to define an environmental profile which best captures the variation in observed detections. Using these reported locations, we can evaluate the local environmental conditions for all of Africa as compared to this theoretical optimal environment for viral presence and evaluate the potential for local zoonotic transmission. We used the methods defined in Pigott et al. to derive data-driven threshold values, which are selected to optimize the tradeoff between accurate classification of known detections and background absences, to classify grid-cells (also referred to as locations) “at-risk” of transmission versus those “not-at-risk” [28, 39]. Given the inherent uncertainty associated with these models, we used different randomly generated dataset subsets to derive a range of threshold values. This allowed us to consider both a more conservative estimate (using a higher threshold at the 95th percentile) and a less conservative estimate (using a lower threshold at the 5th percentile) for all four VHFs in combination with the median estimate used throughout the subsequent analyses. Analyses conducted with these differing thresholds are presented in Additional file 1 (pages 11–14 and Global Health Data Exchange (GHDx)).

### Facility data source

To estimate travel time to health facilities, we used a list of over 95,000 public and private non-profit facilities in sub-Saharan Africa, published by WHO in 2019 [35]. In addition to longitude and latitude and GPS source, this list includes information on administrative units, facility name, facility ownership (governmental, non-governmental organization, public sector, non-profit, among other smaller categories), and type of facility. Noting that there is not one global definition of facility type, the authors retained the types provided in the primary data sources. In order to have less specific categories for our analyses, we broadly recoded the facility types into nine main categories: hospital, health clinic, dispensary, community health unit, health post, health center, maternity ward, medical center, or polyclinic. Terms used in re-categorizing health facility types are available by country in Additional file 1 (pages 21–27).

### Gridded population estimates

To estimate the number of people living in areas with the potential for VHF index cases to arise, gridded population data at a 5 × 5-km resolution were obtained from the WorldPop 2015 database version 2.0 [36]. This dataset was generated using national census data reported at the finest administrative unit, and redistributed per grid-cell using a weighting scale developed using random forest models with land cover, accessibility, night-light, and infrastructure layers, among others, as covariates [40]. Gridded maps were generated for each country for 2010 and were projected at the national level to future years using the 2012 United Nations world urbanization prospects database estimates; total populations estimated were matched up to United Nations national estimates to maintain consistency [41]. These data were interpolated to an annual resolution from a 5-year resolution using an exponential growth rate between the years of the provided data.

### Global friction surface estimates

We used a global friction surface produced by Weiss et al. to quantify how difficult a given 1 × 1-km grid-cell is to navigate in order to calculate travel time to a given facility [37, 42]. This surface considers different datasets of terrains such as bodies of water, elevation, and land cover, as well as means of transportation including roads, railways, and rivers to provide a value of the ease of traversing that grid-cell in minutes of travel via the most efficient method for that terrain—whether by foot, motorized vehicle, or boat (Fig. 1). Infrastructure data, provided from a variety of sources including Open Street Map and Google, defining roads and railways were rasterized to identify which locations matched with these networks. For roads, associated metadata enabled tagging of routes with specific speeds; for railways and water crossings, fixed speeds were assumed. For locations where no road infrastructure was present, speed of movement on foot was evaluated by cross-referencing specific land cover types (derived from MODIS MCD12Q1 imagery [43]), and a questionnaire-derived lookup table of speeds across each category [37]. These speeds were also adjusted to account for topology using imagery-derived elevation assessments [44]. Finally, all speeds were converted from kilometers per hour to minutes required to travel 1 m*.* To quantify the realities of crossing international borders, the friction surfaces add a 1-h penalty to any routes traveled over international borders. Sensitivity analyses looking at walking-only travel can be found in Additional file 1 (page 10).

### Analyses

We focused the analyses on sub-Saharan Africa due to the availability of both VHF suitability maps and facility data. We first quantified the travel time across an “at-risk” grid-cell’s eight nearest neighboring grid-cells, then used least-cost distance methods implemented using Dijkstra’s algorithm to find the shortest pathway from an origin grid-cell of interest (such as a suitable VHF location) to a destination (such as where a facility is present) and provide a cost for navigating that pathway [45]. For these analyses, cost was determined by the resistance and friction between a given grid-cell and every facility (represented as point data) in the country, based upon the navigability of the terrain, to estimate the travel time, in hours, to the most accessible health facility [45–47]. We also calculated country-specific percentile rank of hours of travel for each country. Notably, this was not always the closest facility based on geometric distance due to elevation or other accessibility barriers. In order to analyze the friction data in the context of VHF suitability and the world population layers, both of which exist at a 5 × 5-km resolution, the friction surface data were then aggregated using bilinear interpolation by averaging the four closest grid-cells [48]. Resulting travel times at the 5 × 5-km level were mapped country-wide for each VHF and for an aggregate raster of all VHFs indicating suitability for any of the four pathogens. We mapped each VHF in two ways: first masking out unpopulated areas (defined as fewer than 10 persons per 5 × 5-km grid-cell) and second considering both populated and unpopulated areas in order to articulate the full scope of transmission potential. To determine access to health facilities of different types and presumed different capabilities, we stratified a country’s facilities and recalculated the travel times nationwide.

We were also interested in understanding the proximity of all locations within a country to areas of VHF environmental suitability both within the same country and in neighboring nations. To evaluate this, we estimated the travel time from every grid-cell within a country to the nearest at-risk grid-cell within the same country, as well as the nearest at-risk grid-cell in adjacent countries within a 500-km buffer around the national borders. Due to the challenges associated with quantifying travel time across major bodies of water, large island nations were included in the national health facility accessibility maps but were excluded from all cross-border migration analyses.

These travel time analyses highlight gaps in facility accessibility where long travel times suggest potential misalignment of facilities with populations potentially exposed to VHFs. In light of this potential misalignment, we sought to examine the magnitude both in terms of overall physical access as well as population-weighted physical access, accounting for the reality that some of the regions exhibiting the largest gaps in accessibility are minimally populated or not populated at all, such as regions in or bordering the Sahara. In order to provide insight into these gaps, we explored adding new healthcare resources—such as new facilities or targeted IPC trainings among healthcare workers—in each grid-cell throughout a country and recalculated the country-wide travel times, comparing these to the contemporary assessment; this was replicated for each location to produce a map of mean potential reductions in travel time by location for the entire country. To provide insights on the relative importance of population, we multiplied the travel time in each grid-cell by that grid-cell’s population in order to obtain a “population-weighted” travel time reduction—or time by which each person in the grid-cell would, on average, reduce their travel. All analyses and visualizations were performed using R version 3.5.0 [49].

## Results

For each of the 43 African countries included in these analyses, we enumerate travel times to the most accessible health facilities both in terms of absolute hours of travel (light yellow [0 h] to pink [12 h] to dark purple [24+ h] color palette) and in country-specific percentiles where travel times are scaled based on the minimum and maximum values (yellow [0 to 20%] to teal [40 to 60%] to dark blue [80 to 100%] color palette). We present results for countries we believe most clearly emphasize the utility of a given analysis for preparedness planning; however, Additional file 1 contains profiles for all countries, including pathogen-specific stratification (available on the GHDx).

**Fig. 2:**
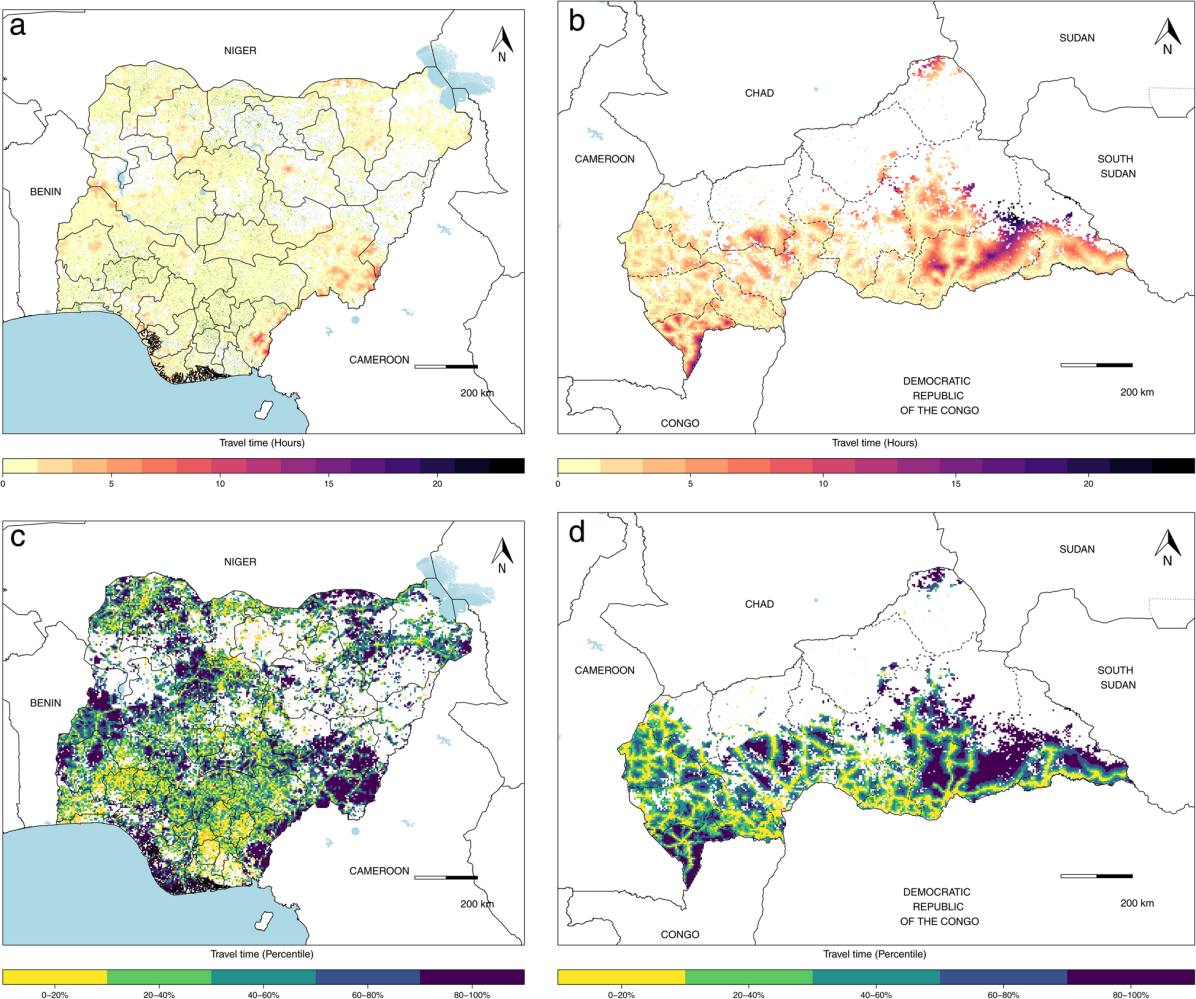
Travel times to health facilities from areas with potential for VHF spillover, Nigeria and the Central African Republic (CAR). **a** The travel times in hours to health facilities in Nigeria, a country with an extensive facility network (green points). **b** The travel time in hours to the more unevenly distributed health facilities (green points) in CAR. **c**, **d** Nationally ranked travel times, with the most remote locations presented in dark blue and the most accessible locations in yellow, with health facilities presented in red. Areas in-country in white have no VHF spillover potential

For each country, we evaluate patterns of in-country facility accessibility for populations at risk for VHFs. In Nigeria, for example, 98% of the population at risk had travel times to a health facility under 2 h, with exceptions in Taraba, Yobe, and Bayelsa states, where travel times were longer (Fig. 2a). The percentile ranked map (Fig. 2c) demonstrates that although the absolute travel times are low for most populations in Nigeria, there are still some locations, including parts of Kaduna and Niger states, where relative physical accessibility to care is lower, providing an insight into where populations may be less connected to health systems. In the Central African Republic (CAR), there are noted disparities in travel times, with 70% of potentially exposed populations in some portions of the country experiencing travel times under 2 h to reach a facility, while others face travel times of 24 h or more (Fig. 2b). This is further highlighted in the percentile ranked map (Fig. 2d), where those areas with high health facility accessibility in absolute time remain among the lower percentiles (in yellow), in contrast to areas with longer absolute travel times which are among the higher 80–100% of the country; this map, in particular, demonstrates the impact of road infrastructure on facility accessibility, visualized with the yellow webbing in Fig. 2d.

**Fig. 3:**
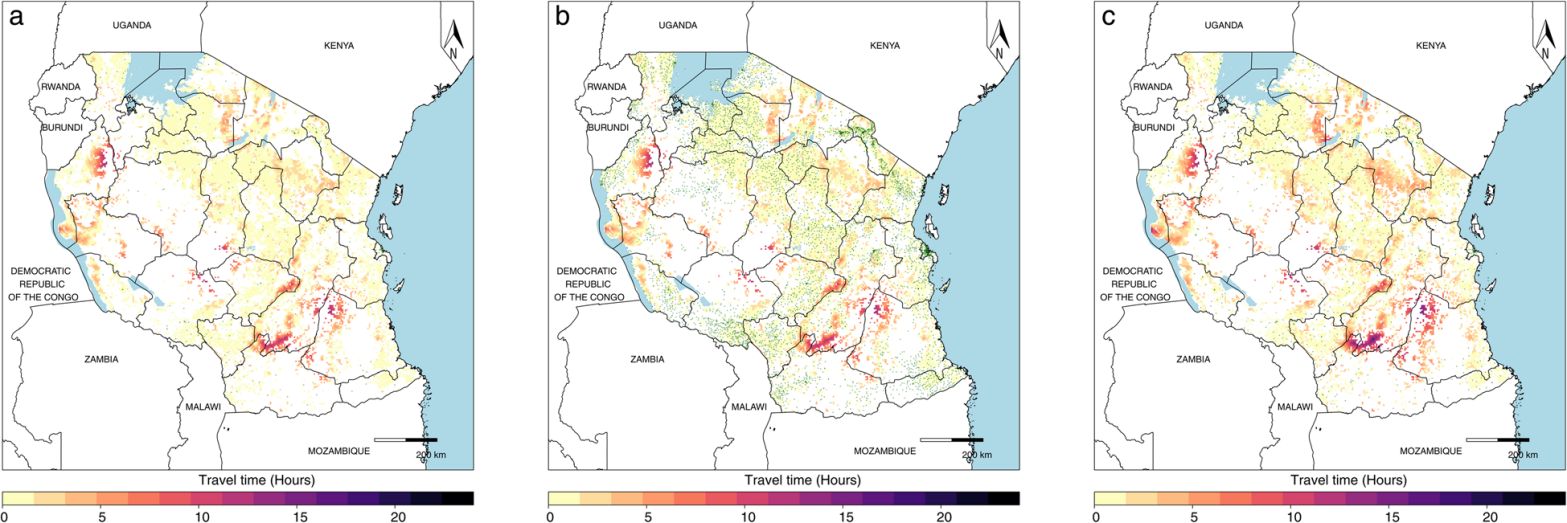
Travel times to Tanzanian health facilities from areas with potential for VHF spillover by facility type. **a** The travel times in hours to hospitals (green points) in Tanzania, **b** the travel time in hours to a health center, and **c** the travel time in hours to a dispensary; areas in-country in white are those without VHF spillover potential. In Tanzania, hospitals were defined as “Designated District Hospital,” “District Hospital,” “National Hospital,” “Referral Hospital,” or “Regional Referral Hospital”; health centers were defined as “Health Center”; and dispensaries were defined as “Dispensary”

When stratified by the type of facility, travel times showed disparate health accessibility profiles, as shown in Tanzania (Fig. 3). While the travel times to the most accessible health facility were largely the same for hospitals (Fig. 3a), as for health centers (Fig. 3b), accessibility differed between the two in certain locations, particularly in the southern part of the Kigoma division on the bank of Lake Tanganyika, where travel times to a hospital exceeded 12 h from some locations versus travel times of 6 to 8 h to health centers. In stark contrast, travel times to both hospitals and health centers were notably longer than those to the nearest dispensary (Fig. 3c), which had a more disperse distribution throughout the country. Stratified analyses by facility type are demonstrated for each country in the country profiles (available on the GHDx). For hospitals present in a given country, we produce a list of those closest to at-risk populations (Additional file 1 page 16 and the GHDx).

**Fig. 4:**
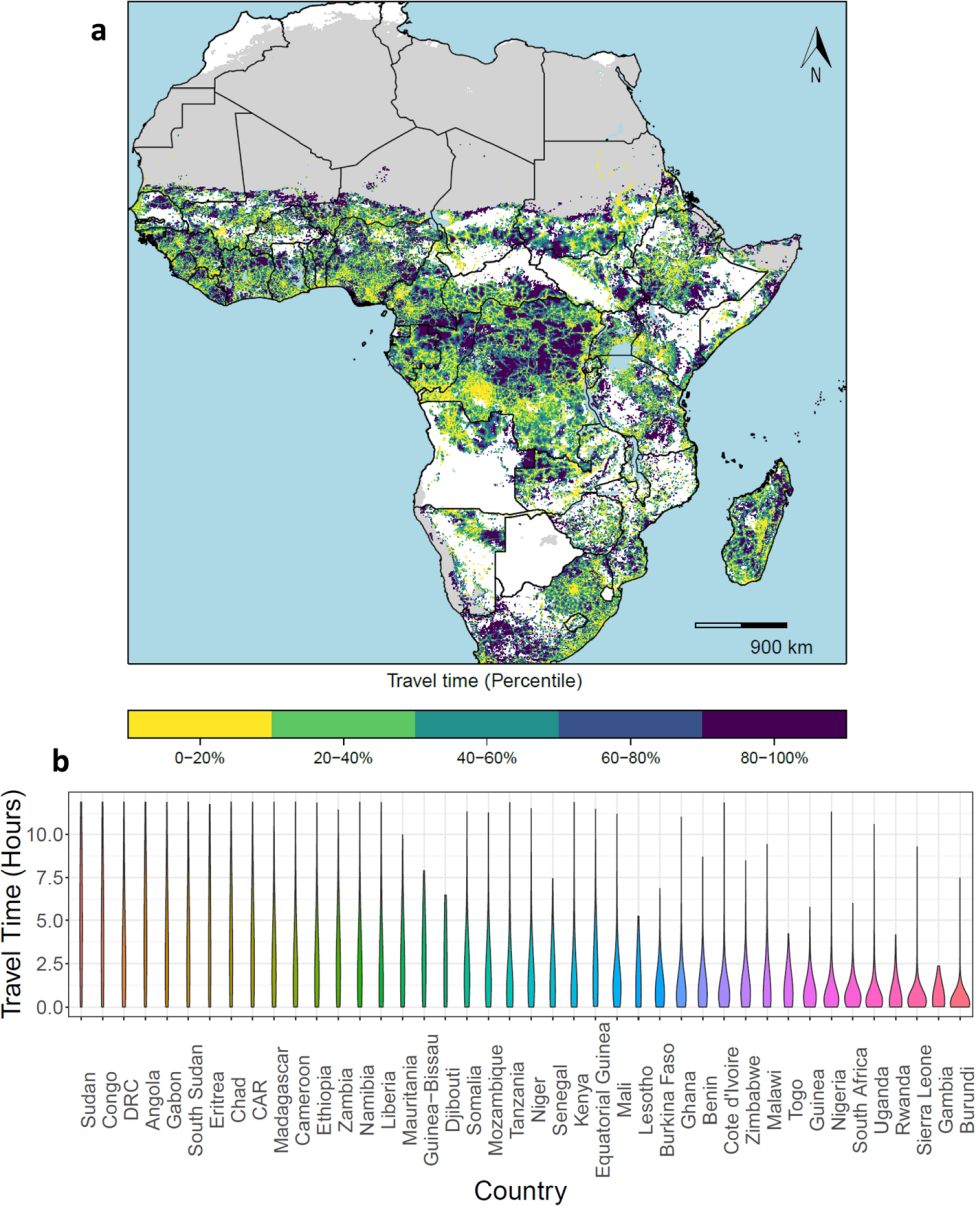
Relative travel times to health facilities from areas with potential for VHF spillover, sub-Saharan Africa. **a** Nationally ranked travel times, with the longest travel times presented in dark blue and the shortest in yellow. Those areas in gray are unpopulated regions, while those areas in white do not have potential for VHF spillover events. **b** The full distribution of travel times within each country, capped at 12 h

When assembled across the African continent, we see relative disparities in VHF-exposed populations’ travel times to health facilities (Fig. 4a). In many cases, long travel times reflect landscape constraints, such as areas within or close to the Sahara in Sudan, Chad, and Mali, or areas of exposure within densely forested regions of the Republic of the Congo. However, in some countries, the relative travel time percentile is indicative of possible misalignment between contemporary health facility provisioning and potential needs (as filtered by our pathogen-set of interest), such as in northwestern Kenya and southern Ethiopia, where populations live in locations proximate to at-risk areas for spillover events, health facilities are sparsely distributed, and navigation challenges exist. We also note where accessibility to health facilities is high regionally, such as across the southern parts of West Africa including Togo, Benin, and Nigeria, suggesting locations to strengthen facilities’ capacities to ensure appropriate treatment and response should a potential VHF case present for care. The distribution of these travel times can be visualized by violin plots (Fig. 4b), where those countries with bottom-heavy figures represent countries where physical access to health facilities is consistently high, whereas those countries with narrow figures represent those countries where physical access is more heterogeneous.

**Fig. 5:**
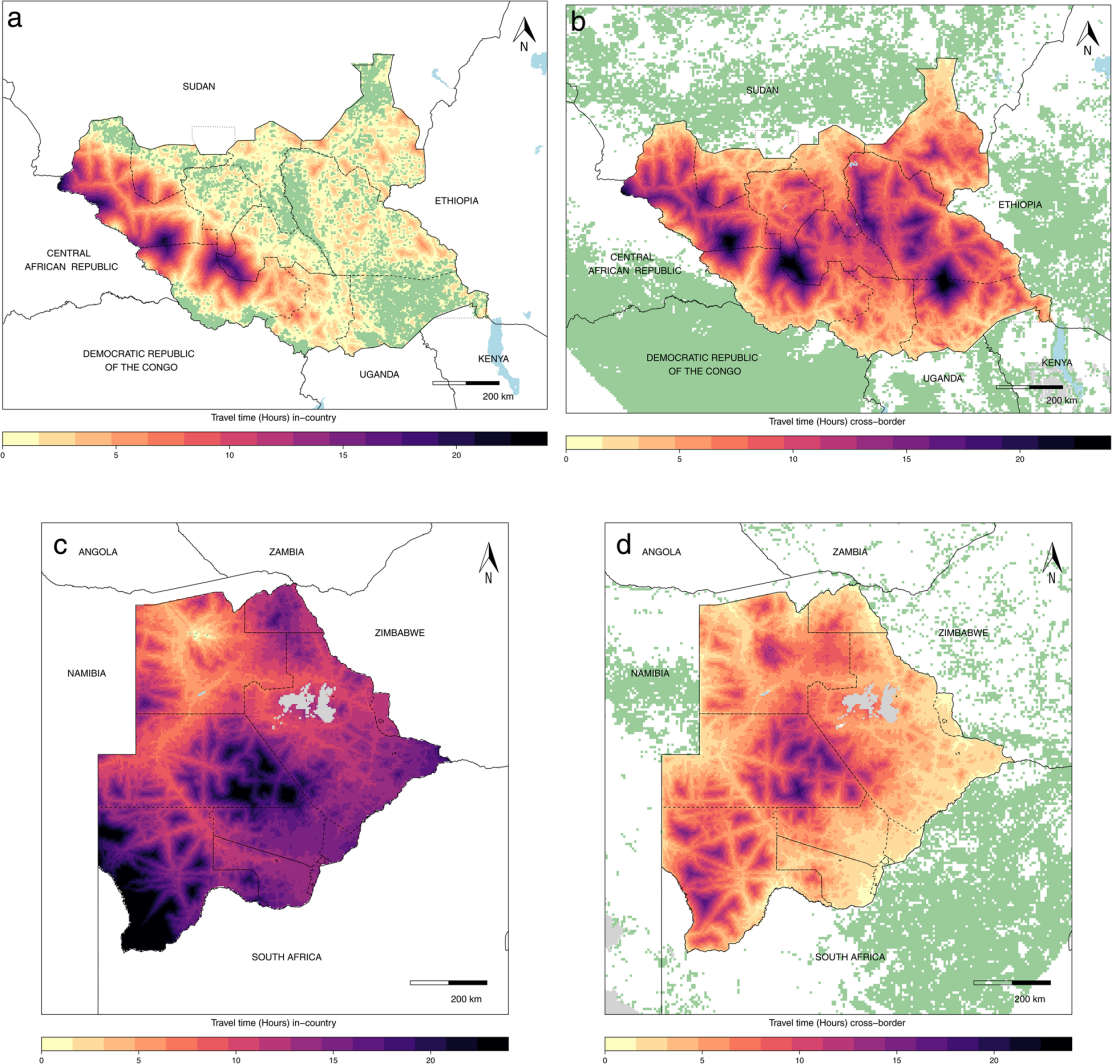
Travel time to locations with VHF spillover event potential in-country and within 500 km of South Sudan and Botswana. **a** The national travel times to reach an area at risk for VHF (in green) in South Sudan, **b** the international locations at risk for VHF (in green) with the travel times it would take to get to a location in South Sudan from those at-risk locations (in green), **c** the internal travel times for Botswana, and **d** the international locations at risk for VHF (in green) for Botswana

In South Sudan, more than half of the at-risk locations in the eastern half of the country are within 2 h of travel to other parts of the country (Fig. 5a), with relatively short travel times to any of the in-country at-risk regions; other locations in the west of the country are comparatively distant from areas suitable for possible spillover of these four VHFs. The cross-border accessibility map (Fig. 5b) paints a similar picture for the western half of the country, although we observed only 4% of the population with travel times within 2 h (including the 1-h cross-border penalty) to international locations suitable for zoonotic transmission versus 79% of populations at risk within 2 h of travel to domestic-only at-risk locations. Figure 5 c and d show that for countries with minimal in-country spillover event potential, such as Botswana where 70% of the population had travel times greater than 12 h to the nearest in-country at-risk location, preparedness plans need to consider neighboring countries as possible sources of infection. Nearly 50% of Botswanans had travel times within 2 h, including the cross-border penalty, to an at-risk location after considering cross-border areas at risk. Given this potential for cross-border transmission, nearby facilities should enhance capacity to detect and treat presenting VHF cases accordingly, particularly in border regions.

**Fig. 6:**
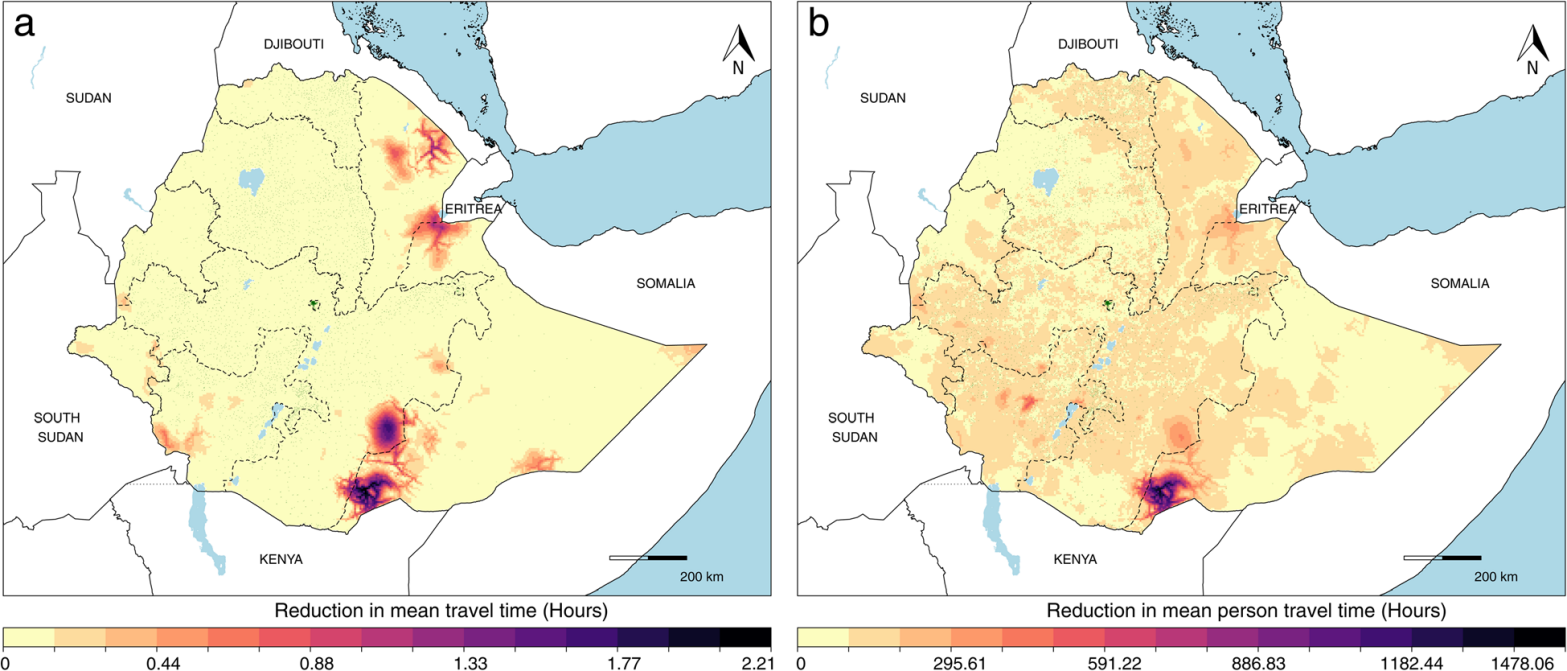
Travel time reductions for new infrastructure placement in Ethiopia. **a** The unweighted reductions in travel time. **b** Population-weighted estimates, with existing health facilities presented as dark green points. **a** Those areas in dark purple represent the locations where travel time would be most reduced based on the current facility landscape and terrain, ignoring population distribution. **b** The areas in dark purple represent the areas where travel time would be most reduced in person-time, thus where the largest per-person reductions would be seen

In Figs. 2, 3, and 4, we demonstrate a country’s health facility accessibility gaps in locations at risk for VHF spillover events and consider the implications of such gaps. One way we contemplated countering such gaps was to consider introducing new resources toward infrastructure and analyze the resulting reductions in overall and person-weighted travel time. Using Ethiopia as an example, in locations such as the forested region near Djibouti, we saw that new infrastructure would result in reductions of approximately 1 h in average travel times (Fig. 6a), but this impact would not be as substantial after accounting for population density. Maximum per-capita access, a priority for preparedness activities, could be increased by focusing new infrastructure to the region along the southern border with Kenya, resulting in travel time reductions of about 30 min per person, for a total of over 300 person-hours, based on both the current facility landscape and the population residing in this region (Fig. 6b).

**Fig. 7:**
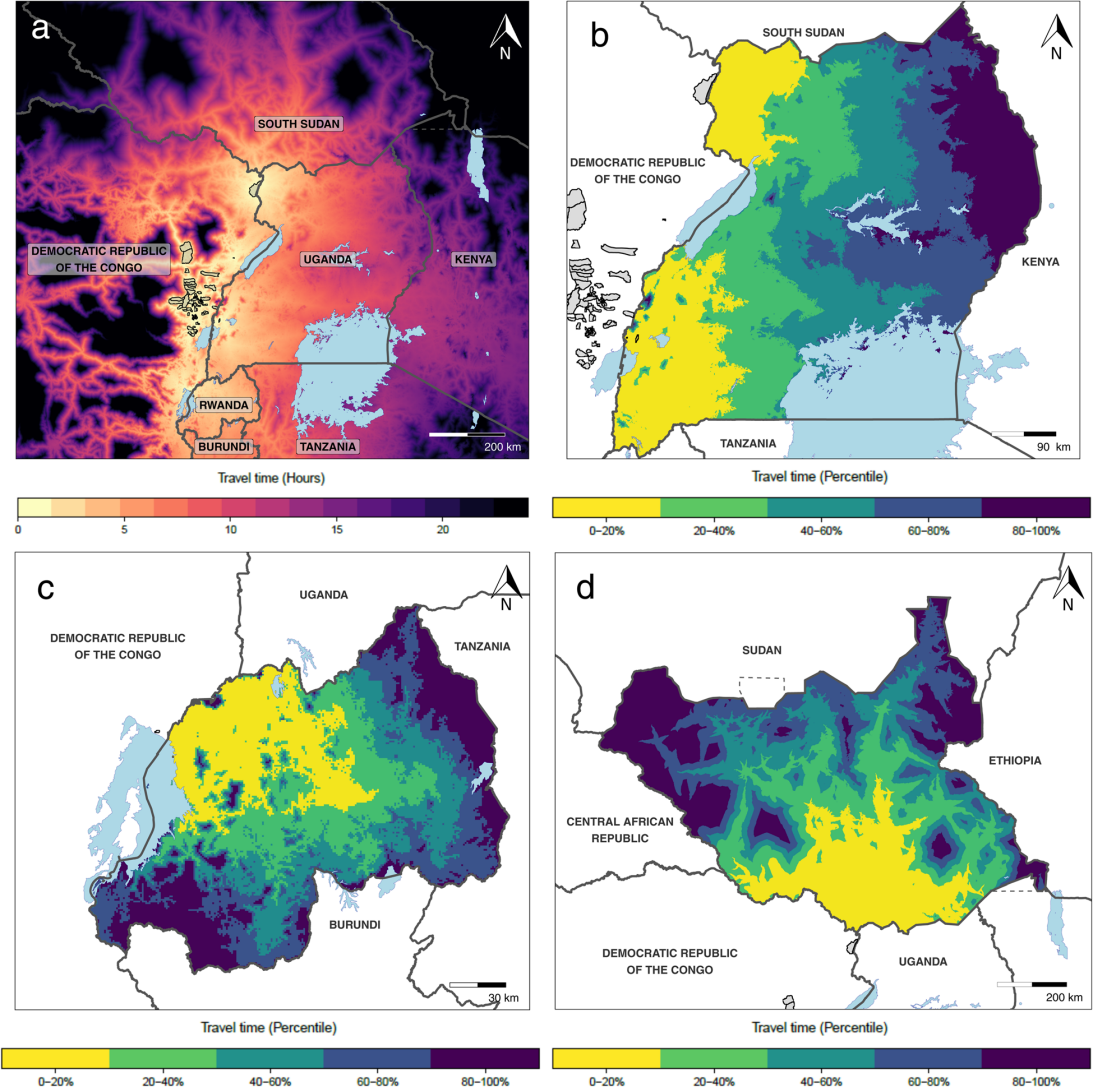
Travel time to locations with recent Ebola cases. **a** The travel time from the DRC and neighboring countries to Ebola-affected areas in DRC and Uganda, **b** the in-country ranked travel times to nearest Ebola case for Uganda, **c** the in-country ranked travel times to nearest Ebola case for Rwanda, and **d** the in-country ranked travel times to nearest Ebola case for South Sudan. Those areas outlined in black in **a** and colored in gray in the other panels are the affected health areas in DRC

While many of our analyses focused on the use of travel time as a pre-emptive preparedness resource, application of these principles is also relevant to mid-outbreak decision-making. Using recent Ebola case data from the ongoing 2018–2019 Ebola outbreak in the Democratic Republic of Congo (DRC) and neighboring Uganda as an example of this application, we estimated travel time to the most accessible Ebola case from locations in eastern DRC, Uganda, Rwanda, and southern South Sudan [50, 51]. Figure 7 a demonstrates several locations in these countries within 4 h of travel from the affected areas, with an estimated population of over 25 million. Similarly, Fig. 7b–d highlights the areas in Uganda, Rwanda, and South Sudan, respectively, with the highest and lowest relative travel times to Ebola cases. As a consequence, we identify the hospitals with the shortest travel times to the nearest Ebola case, and we present the 20 hospitals with the shortest travel times for DRC, Uganda, Rwanda, and South Sudan in Additional file 1 (pages 17–20). Following the detection of Ebola cases in Goma and Ariwara in DRC [50], each of the 4 countries had hospitals within 2 h of the nearest Ebola case, from 2 hospitals in Rwanda to all of the top 20 in DRC.

## Discussion

These analyses provide geospatial insights into preparedness using accessibility as a lens to examine which current health facilities are closest to estimated populations at risk or where gaps in physical coverage of health facilities exist and populations are potentially underserved. Those facilities closest to such at-risk populations should therefore ensure that infrastructure and staff are suitably prepared for patients with suspected VHFs to present for care. As such, we provide a geographic blueprint for considering next steps for improving outbreak preparedness. The resulting maps offer a starting point for country collaboration and uptake, with opportunity for these assessments to be iteratively refined after integrating in-country expertise and local metadata on facility distributions and capabilities. Notably, preparedness activities that would improve alignment of health facilities, and their capacities, to areas with VHF spillover potential would ultimately also improve health systems as a whole [52].

Marked subnational heterogeneities in travel time to health facilities provide insights into needed prioritization and a strategy for implementation of preparedness plans. In areas where travel times are short, such as across much of Nigeria (Fig. 2a), focusing on ensuring a facility’s readiness for detecting and treating VHF cases presenting for care, as well as related prevention and response activities, would be well emphasized given the proximity of potential cases. In locations with long travel times, such as parts of CAR (Fig. 2b), efforts to equally target ensuring populations’ physical accessibility to care as well as the capacity and quality of the facility would be suitable, as these gaps suggest that persons with VHFs may not be identified and served by health systems’ existing health facilities or community responses to reported events. While the maps produced with absolute travel time at the national level highlight differences in physical health facility accessibility, this scale can mask subnational variability, particularly where the absolute travel times are low, as demonstrated in Nigeria, where 90% of travel times were under 2 h (Fig. 2a). Here, the map plotted with travel time in percentiles (Fig. 2c) suggests several locations where physical accessibility is more limited compared to other parts of the country even where absolute travel times are low, such as in parts of Kaduna and Niger states, providing a priority list of locations for improved access. At the continental scale (Fig. 4), we provide a means to systematically survey every country, providing a comprehensive view of vulnerable areas for prioritization and key targets for ensuring existing facilities are capable of quality care. This continental scale is particularly useful for identifying where limited physical accessibility in one country overlaps with limited physical accessibility in neighboring countries, such as where Cameroon, the Republic of the Congo, and CAR all intersect, suggesting regions where VHF cases in any of these countries may not be able to travel easily or quickly to a health facility for care. Conversely, this continental scale also identifies locations where regional travel times to facilities are short, such as in parts of West Africa, emphasizing the need for a facility to be prepared for national or international VHF cases seeking care.

Ensuring the accessibility of healthcare is one step toward providing high-quality, timely treatment and prevention [10]. While our maps show accessibility to any health facility, regardless of capacity, in reality, not every health facility will be prepared for VHF patients presenting for care [9]. Moreover, as part of facility preparedness, facility IPC is essential to prevent sustained transmission [25]. Yet recent studies suggest that competencies and equipment to quickly and effectively isolate VHF patients are often inadequate, such as in the early stages of the West African Ebola outbreak [53], allowing for the possibility of increased institutional secondary transmission. In Tanzania, our maps show the impact of stratifying by the nature of the facility, demonstrating that access to hospitals—where IPC measures are more likely to be in place and presumed capacity to treat VHF patients would be higher—was more limited for the majority of the country when compared to dispensary accessibility (Fig. 3). However, as highlighted in Fig. 1, linkage to health systems may occur at any health facility. Thus, although capacity to provide diagnosis and treatment may not be available at dispensaries, accessing even these smaller facilities would allow for reporting of suspected cases and may trigger a community response as well as hospital referral. While the current facility dataset does not have information regarding facilities’ IPC and treatment readiness, this stratified analysis allows us to begin to understand the accessibility patterns by different types of facilities. This analysis allows us to identify the priority locations where additional resources such as targeted trainings, vaccine stores for future distribution, or additional personal protective equipment, for example, may be most beneficial. Ultimately, in order to provide the most representative health system access maps for country preparedness, close collaboration with country partners is essential for documentation of known health facility and laboratory competencies [52, 54].

Population movement across porous borders was a contributing factor to secondary transmission and disease spread during the 2014–2016 West Africa Ebola outbreak, and emphasizes the need to consider population movement in preparedness plans [30]. For some countries, such as South Sudan (Fig. 5a, b), our work demonstrates that there are both domestic and international risks and that facilities’ and countries’ preparedness plans must consider both pathways. Conversely, in Botswana, for example, while there is limited in-country risk of VHF spillover potential (Fig. 5c), there is considerable connectivity to foreign sites of potential infection to consider (Fig. 5d). While differing in the origin of infection (internal versus international), both scenarios emphasize the potential for secondary transmission and highlight a need for preparedness plans to consider proximity to locations with index case potential, particularly when determining a facility’s readiness to detect, treat, and respond to VHF cases, even in regions or countries where local risk is low. Moreover, these maps provide a guide for identifying vulnerable points of entry beyond known formal channels in order to mitigate international health threats that may otherwise go undetected, a crucial issue to address via National Action Plans [6, 7].

Having identified areas with gaps in health facility accessibility for VHF-exposed populations, we provide an analytical perspective for considering how additional resources directed to healthcare could influence the accessibility landscape as one critical consideration in allocating resources as countries implement National Action Plans [7]. Since most countries have several areas with gaps in health facility accessibility, it is also important to consider population distribution and facility capacity in addressing accessibility. In Ethiopia (Fig. 6), for example, the unweighted reduction maps highlight regions that would reduce overall travel times in the country. However, our population-weighted maps provide the more useful context for considering physical access to new healthcare infrastructure as part of preparedness plans, as these maps depict both the locations where large gaps in accessibility exist as well as the magnitude of the population impacted by such gaps. In the absence of information on each health facility’s capacities, these analyses provide one key component for considering how to allocate resources, particularly with regard to new facility development, but do not address the relationship between physical access and facility capacity of services available. We note that resources directed to supplementing current capacities, bolstering vaccine storage, or expanding community healthcare proficiencies, among other health systems strengthening activities, would also enhance a country’s preparedness.

While the maps of health facility accessibility and in-country and cross-border transmission risks have focused on long-term preparedness planning, many of the key principles are equally applicable to outbreak response and short-term planning operations, highlighting vulnerable communities that might be most at risk within the next phase of the outbreak. As demonstrated for the ongoing Ebola outbreak, these methods can be utilized to inform a variety of action plans, many of which are already identified via the DRC Response Strategy [55]: identifying short-term treatment needs, appropriate targets for ring vaccination, or vulnerable points of entry into neighboring countries. Secondary transmission related to cross-border migration is particularly relevant for the ongoing Ebola outbreak as neighboring Uganda has diagnosed and treated Ebola cases stemming from travel from affected areas within DRC [56]. Similarly, using our travel time estimates, we were able to identify the closest hospitals to the outbreak in DRC and Uganda and neighboring Rwanda and South Sudan (Additional file 1 pages 17–20), identifying key facilities for strengthened capacity to detect, treat, and respond to presenting Ebola cases via targeted trainings, additional protective or laboratory equipment, or supplemental health facility staff in the landscape of an ongoing outbreak. Moreover, as the outbreak progresses, we have been able to identify the hospitals with the shortest travel times to new cases in DRC and neighboring countries, highlighting facilities to prioritize for preparedness activities as the outbreak spreads to more distal locations. As a next step for mid-outbreak preparedness, integrating travel time assessments with mechanistic models predicting near-term spread is an important objective [57].

These analyses and the data are subject to several limitations. First, geographic accessibility is just one facet of equitable care, and we currently lack the ability to assess other factors including quality of care and availability of relevant services. While global assessments of healthcare access and quality have been developed [22], implementing these principles at a local level, for multiple countries simultaneously, has not been addressed. Furthermore, the quantified travel times are not always the actual traveled route to care, as persons may pursue alternative paths to access a health facility, including cross-border travel [58], or chose an alternate facility aside from the closest facility [59, 60], particularly during emergencies [61]. In some instances, while a facility may be available, individuals could already be too ill to travel and are treated at home, or seek non-facility-based forms of healthcare provisioning such as traditional healers [62]. Understanding cultural drivers and barriers to treatment seeking is critical, whether misconceptions of treatment options [63], or fear and stigma associated with conditions [64], as well as recognizing the financial obstacles associated [10]. While a comprehensive continental assessment of these factors is difficult, various geospatial data can be leveraged to act as an indicator of both likelihood to seek care as indicated by household surveys systematically performed across low- and middle-income countries [65] as well as meaningfully demonstrating actual route frequency, as tracked by mobile phones [66]. With this in mind, these current estimates can act only as a general guide, and additional research is required to understand to what extent true humans behave compared to the travel patterns assumed in this study.

Second, three of our four data sources (world population, VHF environmental suitability, and friction surface) are modeled estimates with inherent uncertainty around the presented estimates. We present maps using differing thresholds for the environmental suitability maps in Additional file 1 and note that altering the threshold does impact the number of grid-cells with the potential for local zoonotic transmission, ultimately influencing the resulting travel times to health facilities. It is therefore critical to consider these outputs as a guide for further insights, rather than as definitive results, as the presented results are just one possible scenario among many. Third, these maps utilize environmental features to define potential exposure to pathogens, and do not account for other features that may influence the presence or absence of disease such as the probability of infection following exposure, immunological characteristics of the exposed, behaviors of humans and animals, or vaccination efforts [67]. Fourth, we use one list of geotagged public health facilities in Africa, but note that several health facility lists exist, and that any given list may not be complete, particularly due to the exclusion of private for-profit facilities and specialized facilities, such as prisons or military hospitals. Moreover, the authors noted that although comprehensive, standardized methods were used, data were inconsistently available by country, and therefore, the representativeness of the data in one country is not equivalent to the next, limiting comparability between countries. Additionally, while data on the type of facility exist in the current database, information on the meaning and capacity of each type of facility is not readily available outside of country-specific documentation and differs from one country to the next. Similarly, health facility lists are not static sources, and newly constructed or closed facilities or altered facility capacities could easily affect the accessibility landscape. Thus, working closely with country leadership is essential for producing the most accurate and timely maps for a given country. Last, both the friction surface and the world population layer were generated using data from 2015 and may not reflect current infrastructure, including roads or development, or population changes caused by natural disasters, seasonal patterns, or conflict—a known issue when using static maps for dynamic phenomena [68]. This is particularly relevant to VHFs; for instance, increased VHF transmission has been associated with changes in physical infrastructure (e.g., washed-away roadways) and thus reduced accessibility to health facilities [30].

## Conclusions

Recent trends have demonstrated that pathogens will continue to emerge, both in known settings as well as in new locations. With this study, we show how geospatial analytics can be used to better prepare for outbreaks, whether determining which facilities are closest to potentially exposed populations, where ensuring sufficient infrastructure and staff training is key, or in identifying gaps in health facility accessibility where vulnerable people could currently be underserved. While these analyses provide a guide for sub-Saharan Africa and four VHFs, these methods could be expanded to any country and pathogen, conditional upon data availability. These maps should be considered as one tool among many for assessing subnational misalignment of health systems with VHF environmental suitability and corresponding local outbreak preparedness to enhance resource impact as National Action Plans are updated and implemented.

## Supplementary information

**Additional file 1.** Provides the GATHER checklist, data flow figures depicting the steps for each of the analyses, and Additional file Figures and Tables including: a continental map of the travel time in hours to the most accessible health facility, a sensitivity analysis of the Central African Republic using a friction surface of foot-travel only, examples of uncertainty analyses for each of the four VHF in Cote d’Ivoire, violin plots of the travel times to health facilities for all countries in Africa-both capped at 12 h and unrestricted, a table of the travel times to the nearest locations with VHF spillover event potential from hospitals in Angola, travel times to the most accessible hospital from locations with Ebola cases (2018–2019) in Democratic Republic of the Congo, Uganda, Rwanda, and South Sudan, and, lastly, a list of the facility type specifications and how they were recoded by country. Country profiles for each of the 43 African countries can be found at the “Get Data Files” link on the GHDx record: http://ghdx.healthdata.org/record/ihme-data/travel-time-health-facilities-vhf-outbreak-preparedness-africa. Similarly, all R codes can be found in a zipped folder (IHME_TRAVEL_VHF_2019_CODE.zip) under the files tab.

## Data Availability

All supplemental figures, tables, and code are available at http://ghdx.healthdata.org/record/ihme-data/travel-time-health-facilities-vhf-outbreak-preparedness-africa.

## Abbreviations

CAR: Central African Republic
CCHF: Crimean Congo Hemorrhagic Fever
DRC: Democratic Republic of the Congo
GPS: Global Positioning System
IPC: Infection prevention and control
JEE: Joint External Evaluation
VHF: Viral hemorrhagic fever
WHO: World Health Organization
