# Supplementary material for: Travel time to health facilities in areas of outbreak potential: maps for guiding local preparedness and response
Source: BMC Med. 2019 Dec 30;17:232. doi: 10.1186/s12916-019-1459-6 (PMC6937971; doi:10.1186/s12916-019-1459-6)
Supplement: Supplementary file 1 — Additional file 1. Provides the GATHER checklist, data flow figures depicting the steps for each of the analyses, and Additional file Figures and Tables including: a continental map of the travel time in hours to the most accessible health facility, a sensitivity analysis of the Central African Republic using a friction surface of foot-travel only, examples of uncertainty analyses for each of the four VHF in Cote d’Ivoire, violin plots of the travel times to health facilities for all countries in Africa-both capped at 12 h and unrestricted, a table of the travel times to the nearest locations with VHF spillover event potential from hospitals in Angola, travel times to the most accessible hospital from locations with Ebola cases (2018–2019) in Democratic Republic of the Congo, Uganda, Rwanda, and South Sudan, and, lastly, a list of the facility type specifications and how they were recoded by country. Country profiles for each of the 43 African countries can be found at the “Get Data Files” link on the GHDx record: http://ghdx.healthdata.org/record/ihme-data/travel-time-health-facilities-vhf-outbreak-preparedness-africa. Similarly, all R codes can be found in a zipped folder (IHME_TRAVEL_VHF_2019_CODE.zip) under the files tab. [file 12916_2019_1459_MOESM1_ESM.docx]

Additional File 1 for “**Travel time to health facilities in areas of viral hemorrhagic fever outbreak potential: maps for guiding local preparedness and response**”

Table of Contents

**Additional Figures2**

**Additional Tables3**

**1.0 GATHER compliance4**

**2.0 Methodology6**

**3.0 Additional Results 9**

Additional Figures

Figure S1 Data flow for travel time to most accessible hospital for areas with VHF spillover potential 6

Figure S2 Data flow for travel time to any grid-cell at risk for VHF spillover from any other location 7

Figure S3 Data flow for reductions in travel time via new resource allocation 8

Figure S4 Absolute travel times to health facilities from areas with potential for VHF spillover, sub-Saharan Africa9

Figure S5 Travel times to the most accessible health facility by foot for Central African Republic 10

Figure S6 Maps of the uncertainty around the Ebola environmental suitability niche maps for Cote d’Ivoire 11

Figure S7 Maps of the uncertainty around the Marburg environmental suitability niche maps for Cote d’Ivoire 12

Figure S8 Maps of the uncertainty around the CCHF environmental suitability niche maps for Cote d’Ivoire13

Figure S9 Maps of the uncertainty around the CCHF environmental suitability niche maps for Cote d’Ivoire14

Figure S10 Violin plots of the distribution of travel times (in hours) from areas with VHF spillover event potential, by country 15

Additional Tables

Table S1 Guidelines for Accurate and Transparent Health Estimates Reporting (GATHER) checklist4

Table S2 Travel time to most accessible hospital from locations with VHF spillover event potential, Angola 16

Table S3 Travel time to most accessible hospital from locations with Ebola cases (2018-2019), Democratic Republic of the Congo17

Table S4 Travel time to most accessible hospital from locations with Ebola cases (2018-2019), Uganda 18

Table S5 Travel time to most accessible hospital from locations with Ebola cases (2018-2019), Rwanda 19

Table S6 Travel time to most accessible hospital from locations with Ebola cases (2018-2019), South Sudan 20

Table S7 Terms used for facility stratification by country 21

1. GATHER Compliance

**Table S1.** Guidelines for Accurate and Transparent Health Estimates Reporting (GATHER) checklist.

| **Item #** | **Checklist item** | **Reported on page #** |
| --- | --- | --- |
| **Objectives and Funding** | | |
| **1** | Define the indicator(s), populations (including age, sex, and geographic entities), and time period(s) for which estimates were made. | Main text: Introduction |
| **2** | List the funding sources for the work. | Main text: Acknowledgements |
| **Data Inputs** | | |
| *For all data inputs from multiple sources that are synthesized as part of the study:* | | |
| **3** | Describe how the data were identified and how the data were accessed. | Main text: Methods (data sources) pages 9-11 Additional File: Additional Figure S1 page 6 |
| **4** | Specify the inclusion and exclusion criteria. Identify all ad-hoc exclusions. | Main text: Methods (data sources) pages 9-11  Additional File: Additional Figure S1 page 6 |
| **5** | Provide information on all included data sources and their main characteristics. For each data source used, report reference information or contact name/institution, population represented, data collection method, year(s) of data collection, sex and age range, diagnostic criteria or measurement method, and sample size, as relevant. | Main text: Methods (data sources) pages 9-11  Additional File: Additional Figure S1 page 6 |
| **6** | Identify and describe any categories of input data that have potentially important biases (e.g., based on characteristics listed in item 5). | Main text: Discussion pages 19-25 |
| *For data inputs that contribute to the analysis but were not synthesized as part of the study:* | | |
| **7** | Describe and give sources for any other data inputs. | NA |
| *For all data inputs:* | | |
| **8** | Provide all data inputs in a file format from which data can be efficiently extracted (e.g., a spreadsheet rather than a PDF), including all relevant meta-data listed in item 5. For any data inputs that cannot be shared because of ethical or legal reasons, such as third-party ownership, provide a contact name or the name of the institution that retains the right to the data. | http://ghdx.healthdata.org/record/ihme-data/travel-time-health-facilities-vhf-outbreak-preparedness-africa |
| **9** | Provide a conceptual overview of the data analysis method. A diagram may be helpful. | Main text: Methods (data sources) pages 9-11  Additional File: Additional Figure S1 page 6 |
| **10** | Provide a detailed description of all steps of the analysis, including mathematical formulae. This description should cover, as relevant, data cleaning, data pre-processing, data adjustments and weighting of data sources, and mathematical or statistical model(s). | Main text: Methods (data sources) pages 9-11 |
| **11** | Describe how candidate models were evaluated and how the final model(s) were selected. | NA |
| **12** | Provide the results of an evaluation of model performance, if done, as well as the results of any relevant sensitivity analysis. | Additional File: Additional Figure S1 page 6 |
| **13** | Describe methods for calculating uncertainty of the estimates. State which sources of uncertainty were, and were not, accounted for in the uncertainty analysis. | Main text: Methods (data sources) pages 9-11  Additional File: Additional Figure S1 page 6 |
| **14** | State how analytic or statistical source code used to generate estimates can be accessed. | http://ghdx.healthdata.org/record/ihme-data/travel-time-health-facilities-vhf-outbreak-preparedness-africa |
| **Results and Discussion** | | |
| **15** | Provide published estimates in a file format from which data can be efficiently extracted. | http://ghdx.healthdata.org/record/ihme-data/travel-time-health-facilities-vhf-outbreak-preparedness-africa |
| **16** | Report a quantitative measure of the uncertainty of the estimates (e.g. uncertainty intervals). | Additional Information:  Additional Figures S6-S9 pages 11-14  http://ghdx.healthdata.org/record/ihme-data/travel-time-health-facilities-vhf-outbreak-preparedness-africa |
| **17** | Interpret results in light of existing evidence. If updating a previous set of estimates, describe the reasons for changes in estimates. | Main text: Results pages 14-18 |
| **18** | Discuss limitations of the estimates. Include a discussion of any modelling assumptions or data limitations that affect interpretation of the estimates. | Main text: Results pages 17-18 |

1. Methodology

**
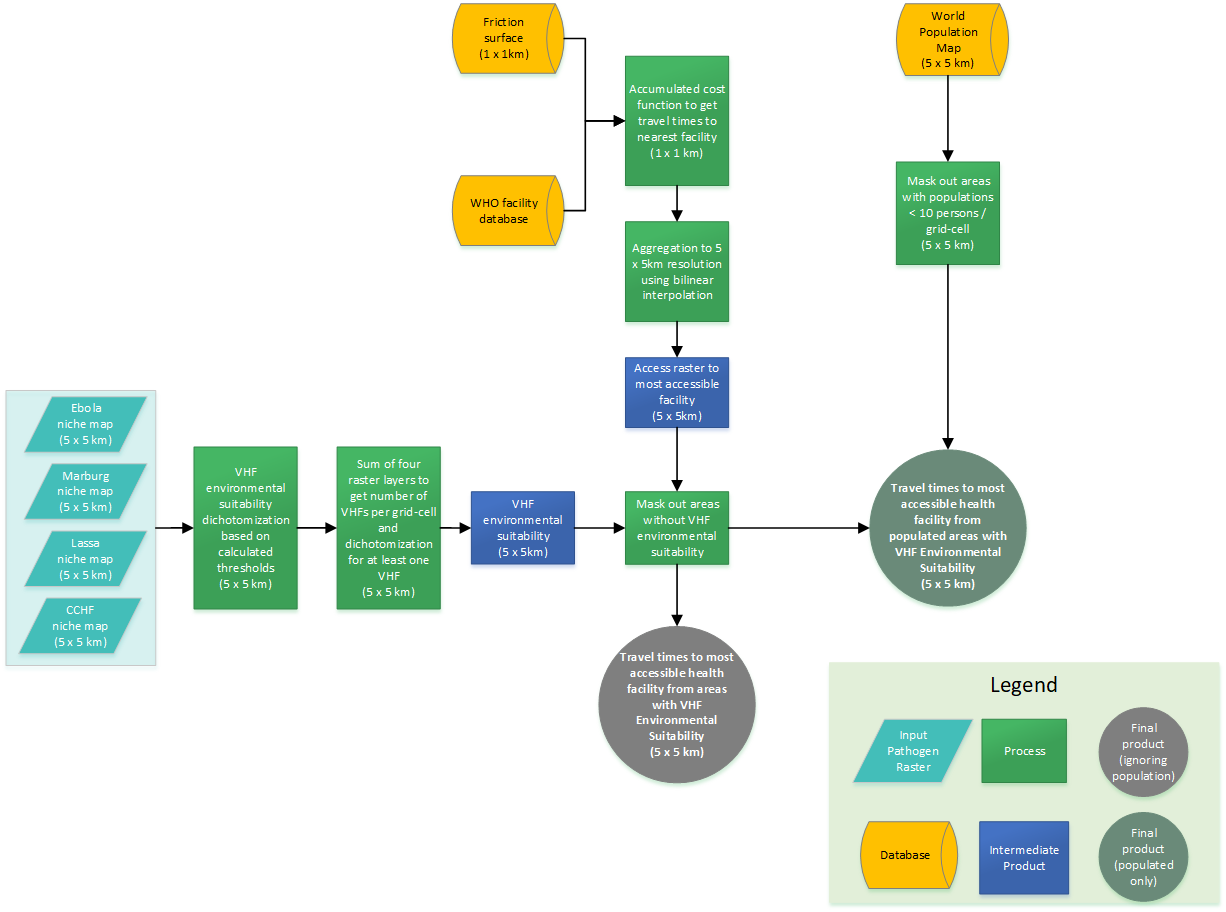
Figure S1. Data flow for travel time to most accessible hospital for areas with VHF spillover potential.** This data flow visualization demonstrates the flow of data from ecological niche maps for the four viral hemorrhagic fevers (light blue parallelograms) through the incorporation of the other three data sources (WHO health facilities, WorldPop raster layer, and the Malaria Atlas Project’s friction surface, in yellow) and through raster manipulation to the final product of a raster of travel times to the nearest health facility from locations with VHF spillover potential, both overall (grey circle) and masking out un-populated areas (green circle).


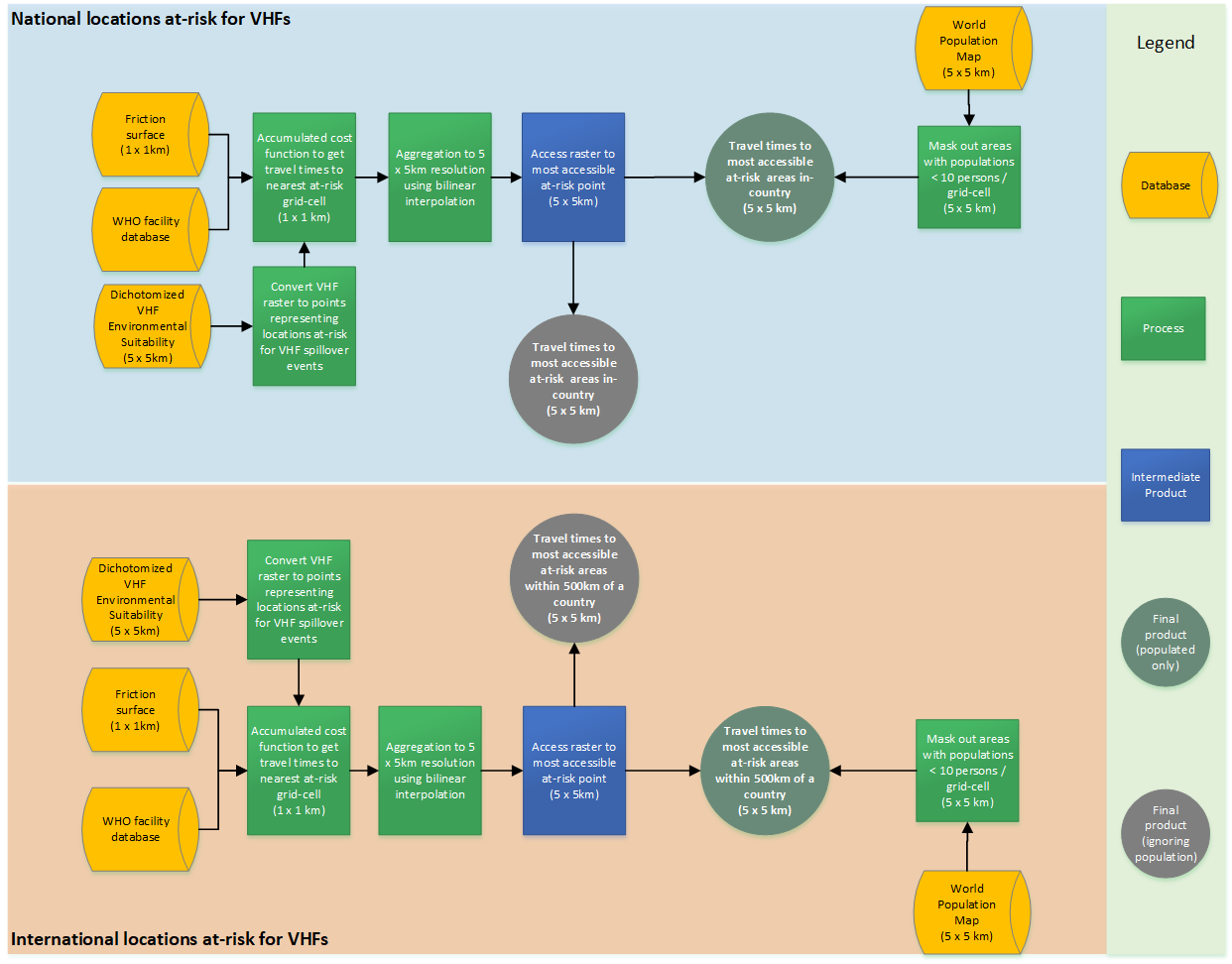
**Figure S2. Data flow for travel time to any grid-cell at risk for VHF spillover from any other location.** This data flow visualization demonstrates the flow of data from three data sources (dichotomized ecological niche maps for four VHFs, WorldPop raster layer, and the Malaria Atlas Project’s friction surface, in yellow) and through raster manipulation to the final product of a raster of travel times to the nearest at-risk grid-cell from locations not at-risk both overall (grey circle) and masking out un-populated areas (green circle). The top half of the figure (shaded blue) demonstrates times to at-risk grids in-country, while the bottom half (shaded orange) represents times to at-risk grids within 500km of a country’s borders.

**Figure S3. Data flow for reductions in travel time via new resource allocation.** This data flow visualization demonstrates the flow of data to produce the travel time reduction maps. The Malaria Atlas Project’s friction surface and WHO facility database (yellow databases) are combined to produce the accumulated cost surface, which is then aggregated to 5 x 5 km resolution, and converted to a surface of points. We sequentially place new infrastructure in each of these grid-cells, and recalculate travel times to the most accessible facility for each new point. For the unweighted map, we then subtract this assessment, with areas without VHF spillover event potential masked out, from the contemporary assessment to get the mean reduction in travel time to new infrastructure. For the weighted map, we multiply the travel times by the population in that grid-cell to get a map of the mean population-weighted reduction in travel times.


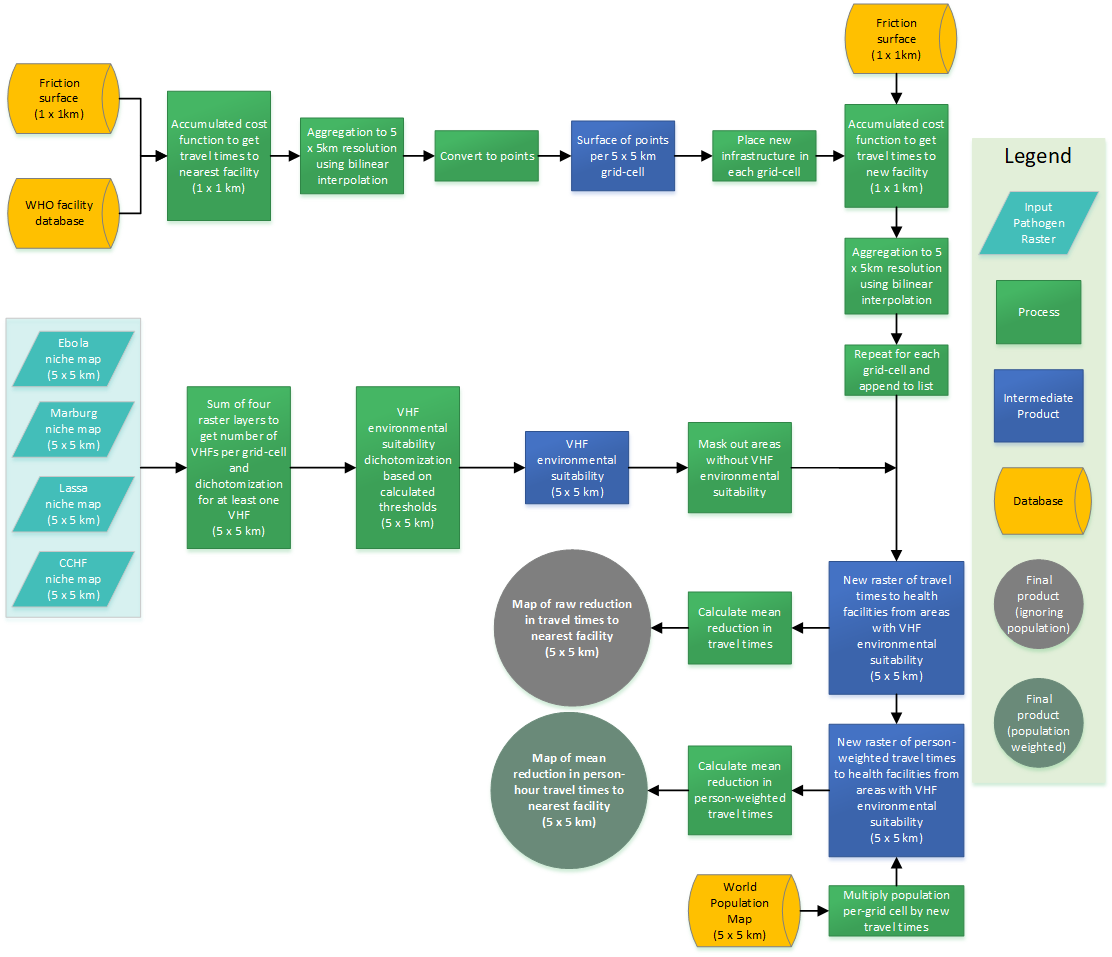


1. Supplementary Material


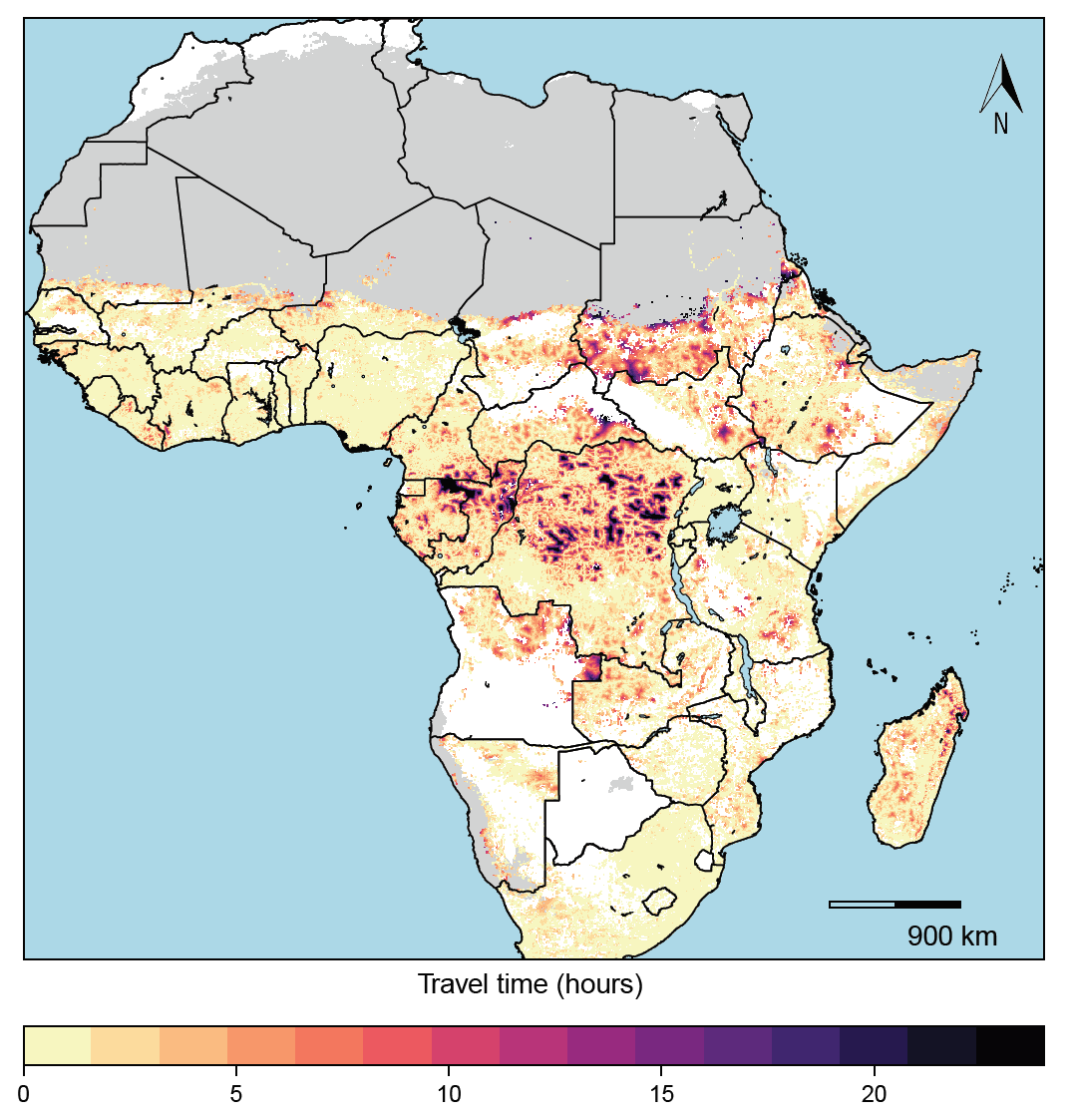
**Figure S4. Absolute travel times to health facilities from areas with potential for VHF spillover, sub-Saharan Africa.**This figure portrays absolute travel times with the longest travel times presented in dark purple and the shortest travel times in light yellow. Those areas in grey are unpopulated regions while those areas in white do not have potential for VHF spillover events.


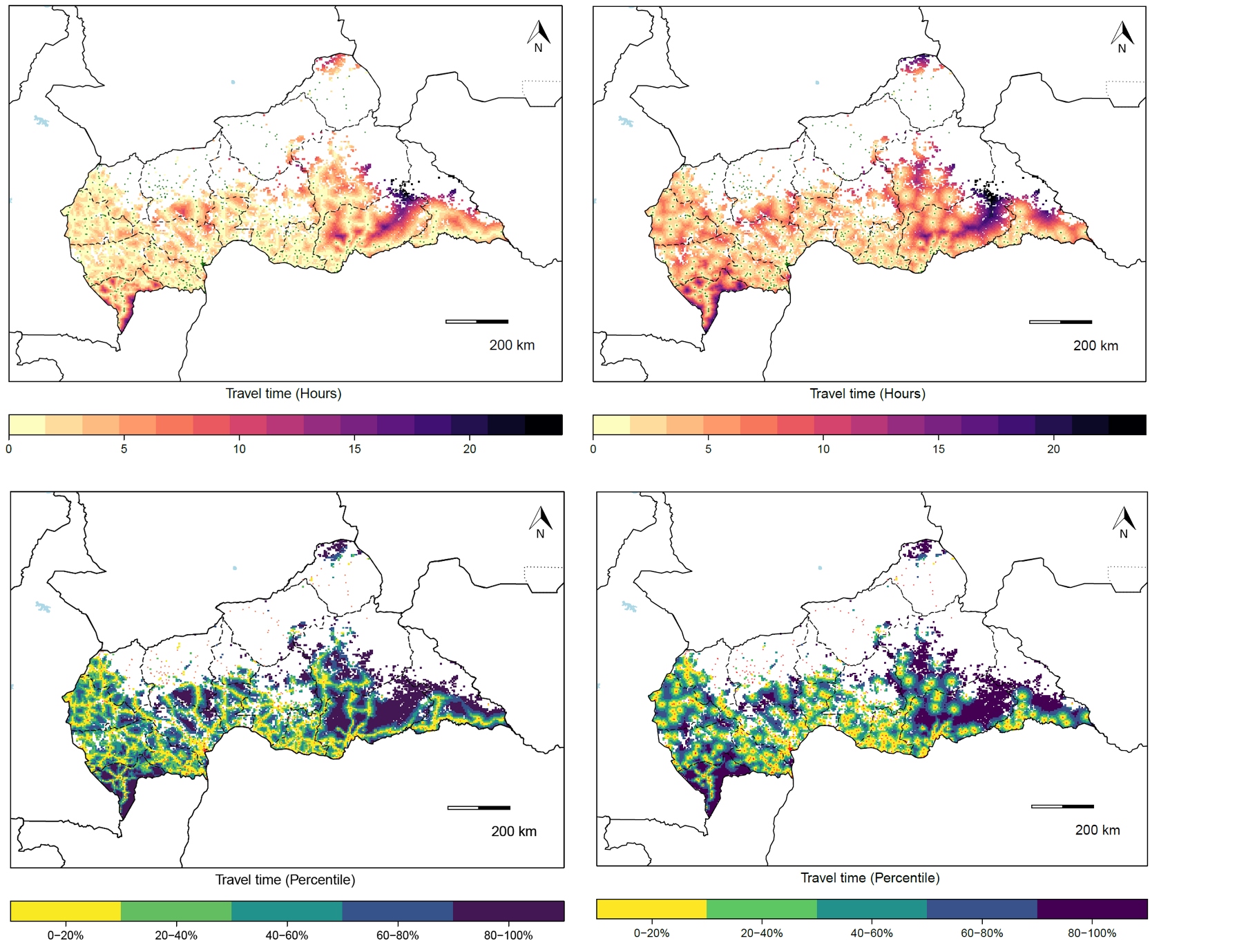
**Figure S5. Travel times to the most accessible health facility by foot for Central African Republic.** Panel a) presents the results of the assessment of the travel time to health facilities using the original friction layer which presents the absolute travel times using the fastest available means of travel while panel b) presents the absolute travel times using the foot-travel only layer. Panel c) presents the results of the assessment of percentile-ranked travel times using the original friction layer, while panel d) presents the percentile-ranked travel times using the foot-travel only layer.

**d**

**c**

**b**

**a**

**Figure S6. Maps of the uncertainty around the Ebola environmental suitability niche maps for Cote d’Ivoire.** The first panel (a) demonstrates the travel times to health facilities from areas with spillover event potential using the most conservative 95^th^ percentile (0.468) as a threshold for classifying whether a grid-cell has spillover event potential, panel (b) uses the median (0.349) as the threshold, and panel (c) uses the least conservative 5^th^ percentile (0.210) as the threshold.


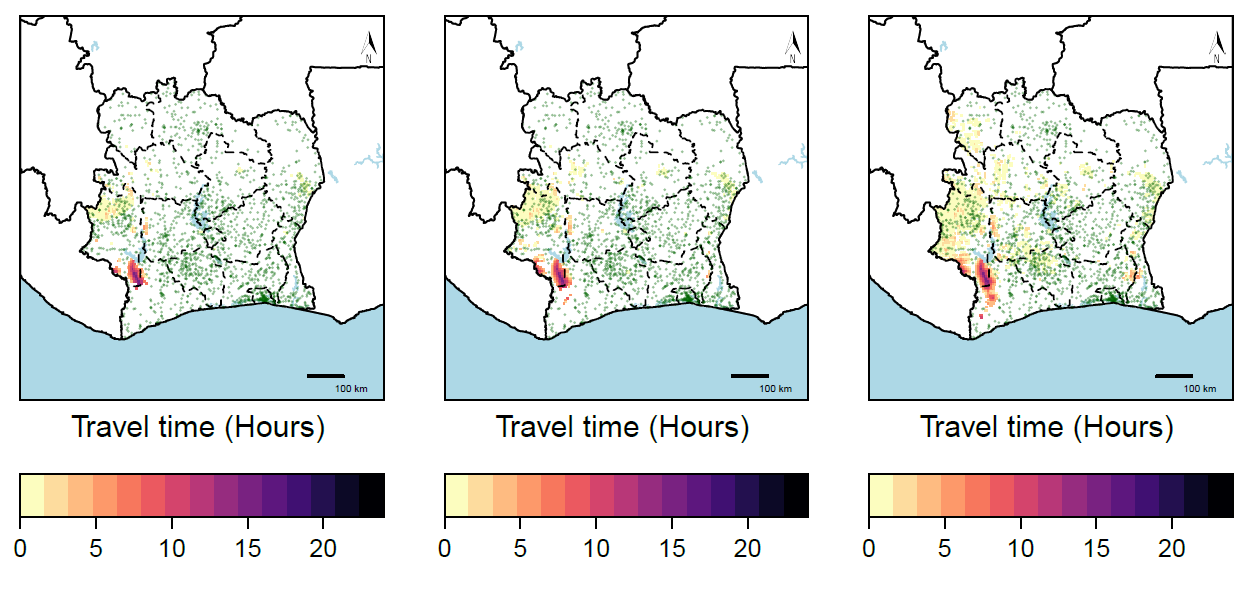


**c**

**b**

**a**


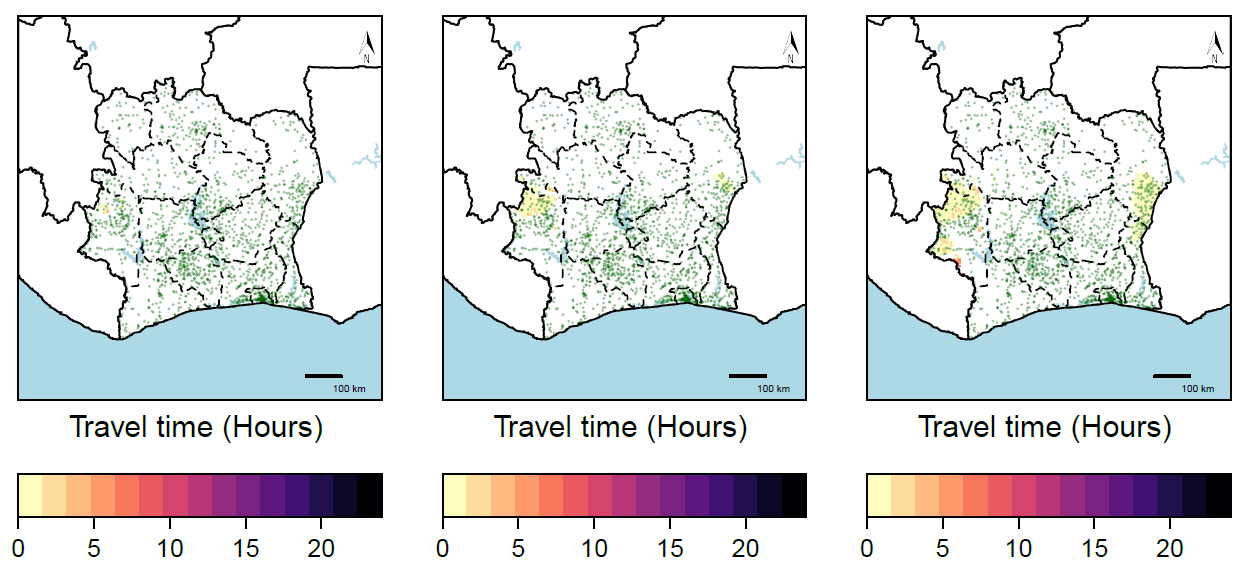
**Figure S7. Maps of the uncertainty around the Marburg environmental suitability niche maps for Cote d’Ivoire.** The first panel (a) demonstrates the travel times to health facilities from areas with spillover event potential using the most conservative 95^th^ percentile (0.608) as a threshold for classifying whether a grid-cell has spillover event potential, panel (b) uses the median (0.399) as the threshold, and panel (c) uses the least conservative 5^th^ percentile (0.300) as the threshold.

**c**

**b**

**a**


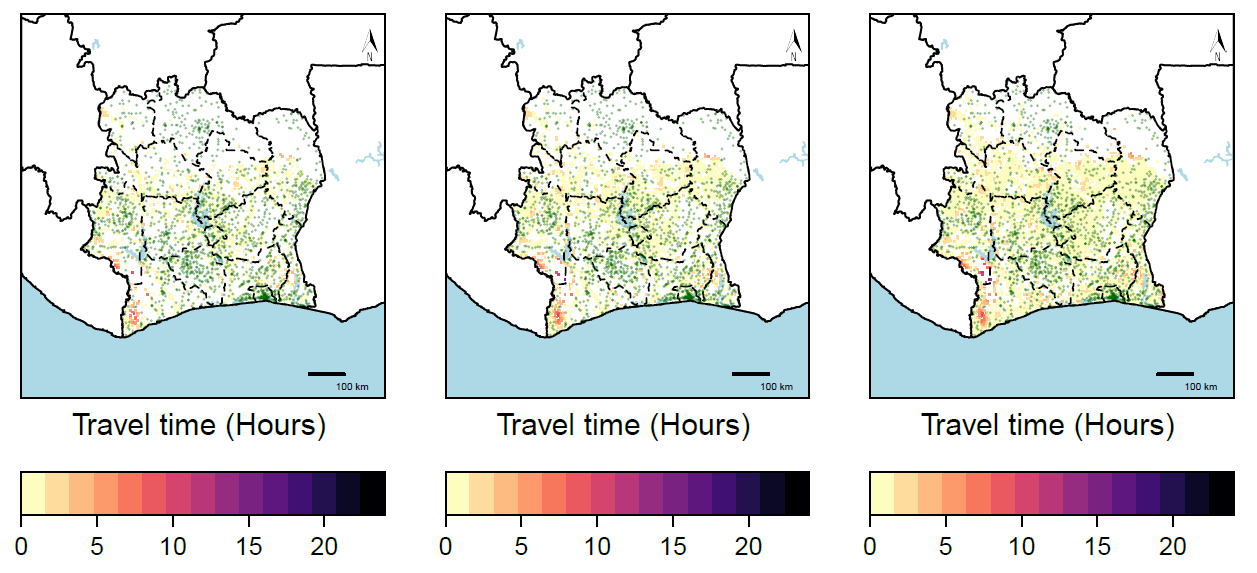
**Figure S8. Maps of the uncertainty around the CCHF environmental suitability niche maps for Cote d’Ivoire.** The first panel (a) demonstrates the travel times to health facilities from areas with spillover event potential using the most conservative 95^th^ percentile (0.025) as a threshold for classifying whether a grid-cell has spillover event potential, panel (b) uses the median (0.019) as the threshold, and panel (c) uses the least conservative 5^th^ percentile (0.014) as the threshold.

**c**

**b**

**a**


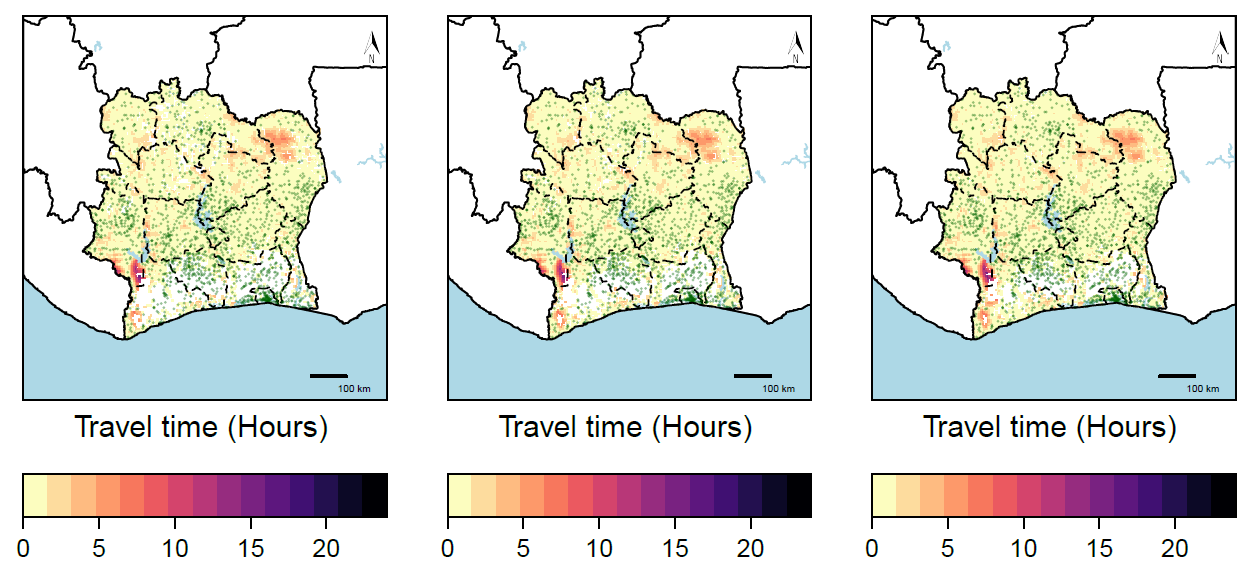
**Figure S9. Maps of the uncertainty around the Lassa environmental suitability niche maps for Cote d’Ivoire.** The first panel (a) demonstrates the travel times to health facilities from areas with spillover event potential using the most conservative 95^th^ percentile (0.443) as a threshold for classifying whether a grid-cell has spillover event potential, panel (b) uses the median (0.396) as the threshold, and panel (c) uses the least conservative 5^th^ percentile (0.339) as the threshold.

**c**

**b**

**a**


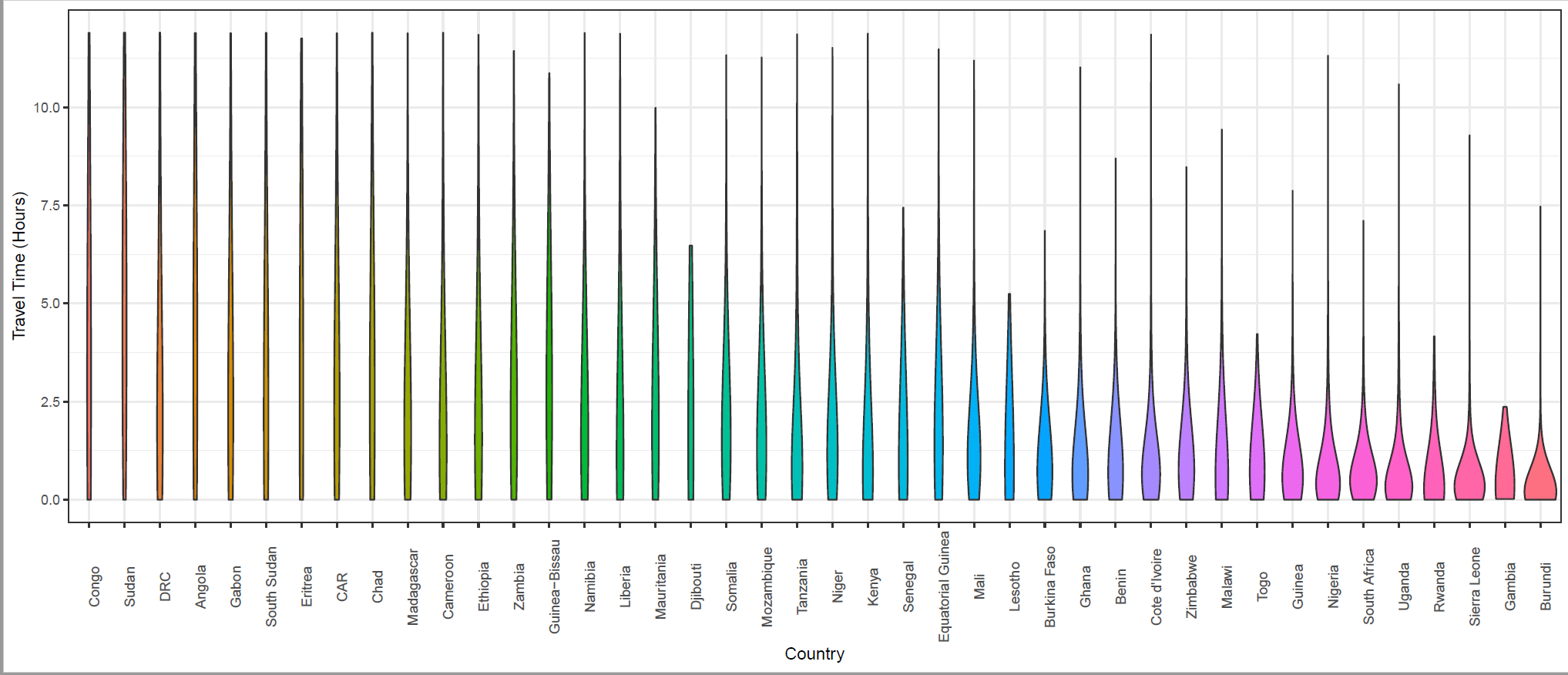

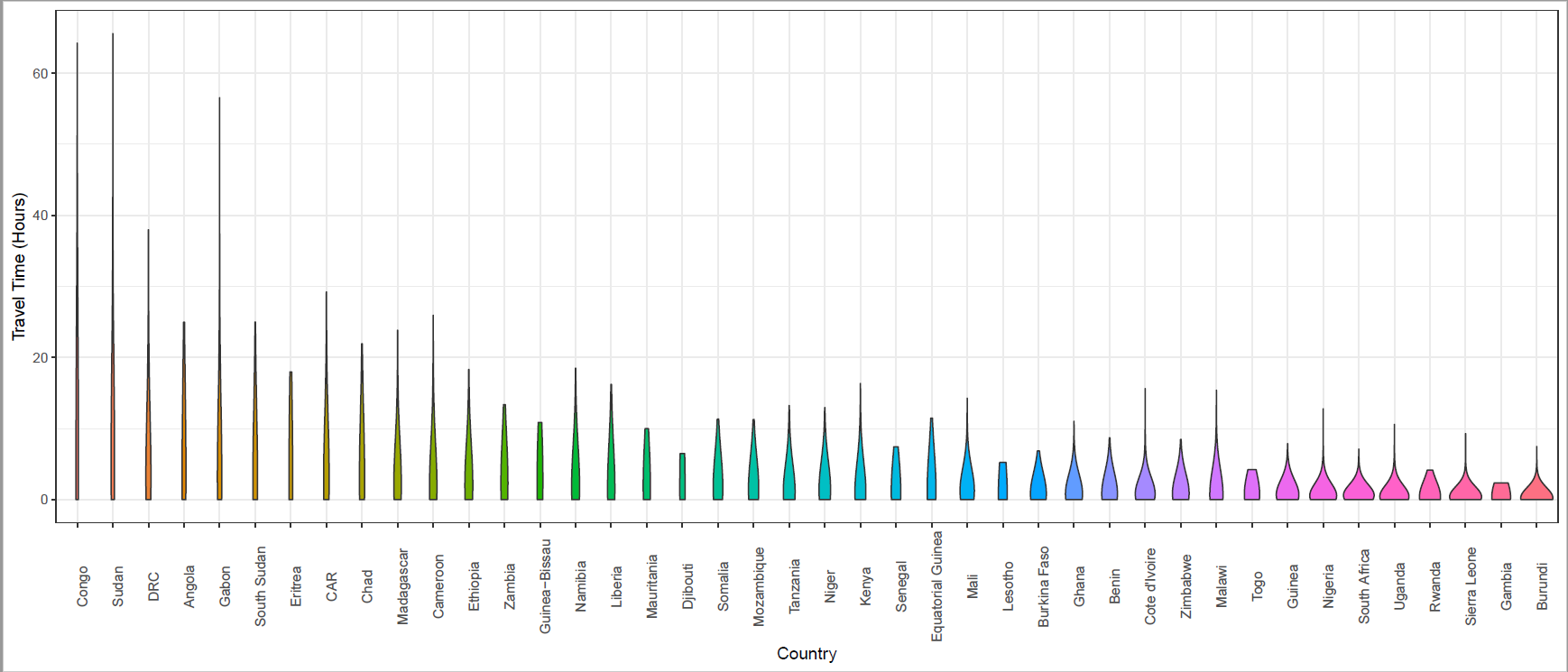
**Figure S10. Violin plots of the distribution of travel times (in hours) from areas with VHF spillover event potential, by country.** Panel a) presents the un-trimmed travel times by country while panel b) restricts the maximum travel time to 12+ hours to more closely evaluate the distributions in under one half-day of travel.

**B**

**A**

**Table S2 Travel time to nearest locations with VHF spillover event potential from all hospitals in Angola.** This table presents the travel times to a location with VHF spillover event potential from hospitals (with their first administrative unit in parentheses) in rank order, colored by hours of travel.
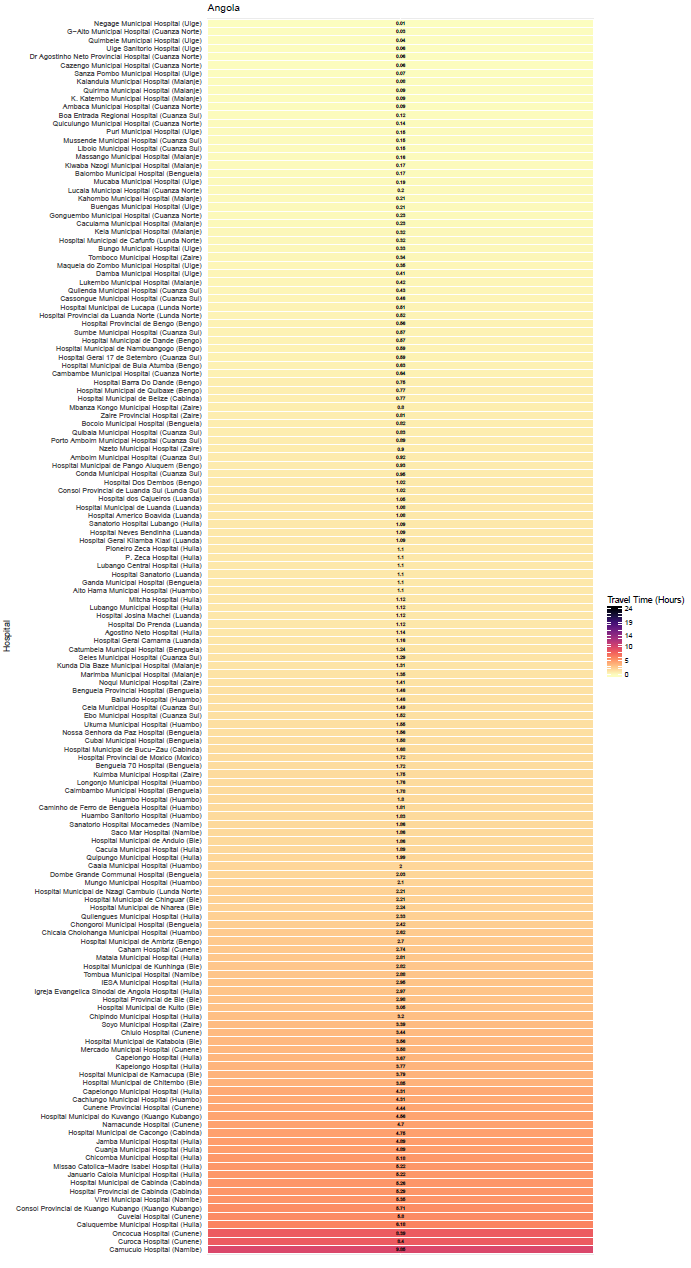


**Table S3 Travel time to most accessible hospital from locations with Ebola cases (2018-2019), Democratic Republic of the Congo.** This table presents the travel times from the twenty closest hospitals in the Democratic Republic of the Congo to a location with Ebola cases based on case data from the WHO’s Situation Report 50, published July 16, 2019. The hospitals are presented in rank-order from closest (shortest travel times) to furthest (longest travel time) among the twenty.


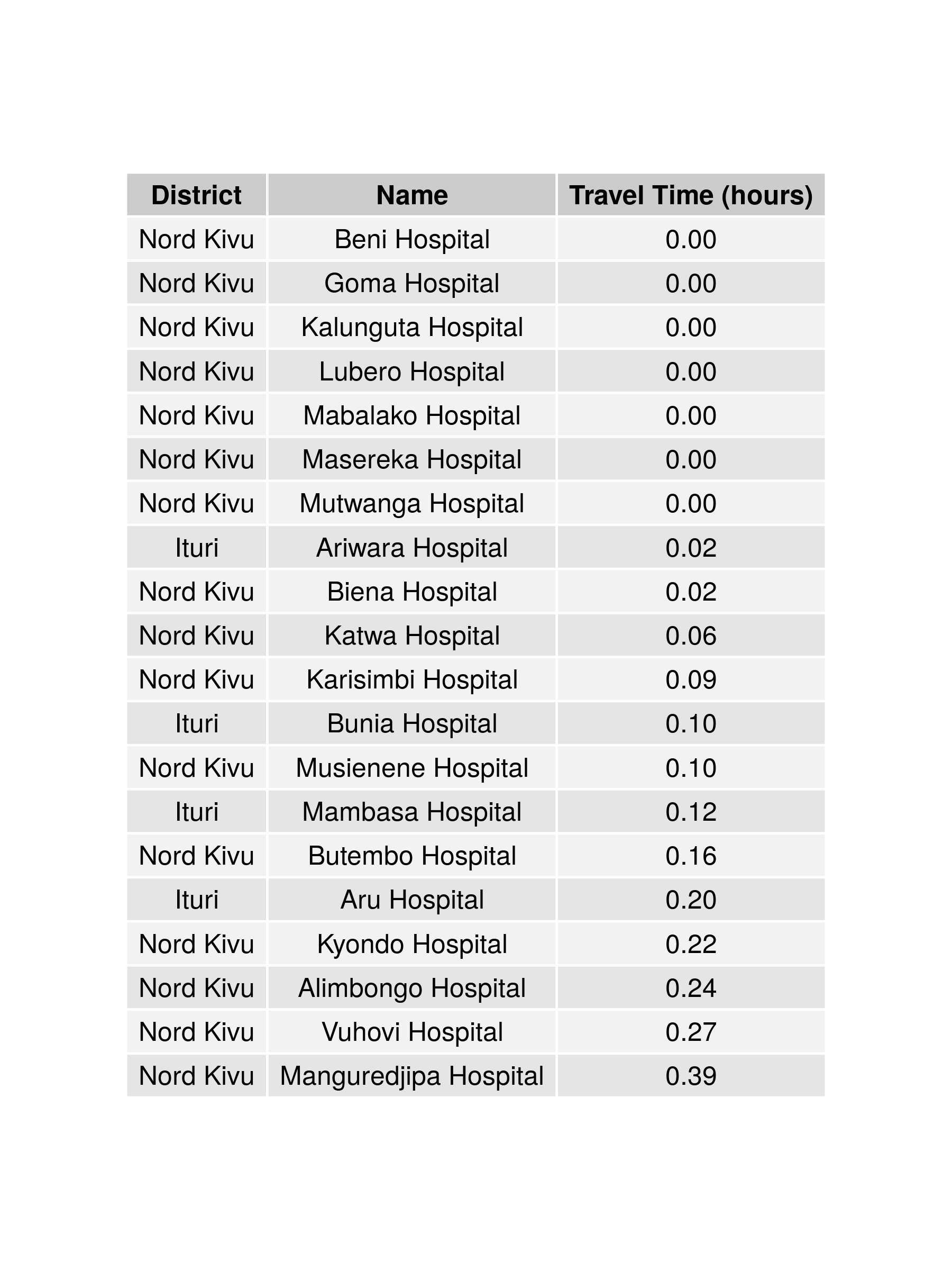


**Table S4. Travel time to most accessible hospital from locations with Ebola cases (2018-2019), Uganda.** This table presents the travel times from the twenty closest hospitals in Uganda to a location with Ebola cases based on case data from the WHO’s Situation Report 50, published July 16, 2019. The hospitals are presented in rank-order from closest (shortest travel times) to furthest (longest travel time) among the twenty.


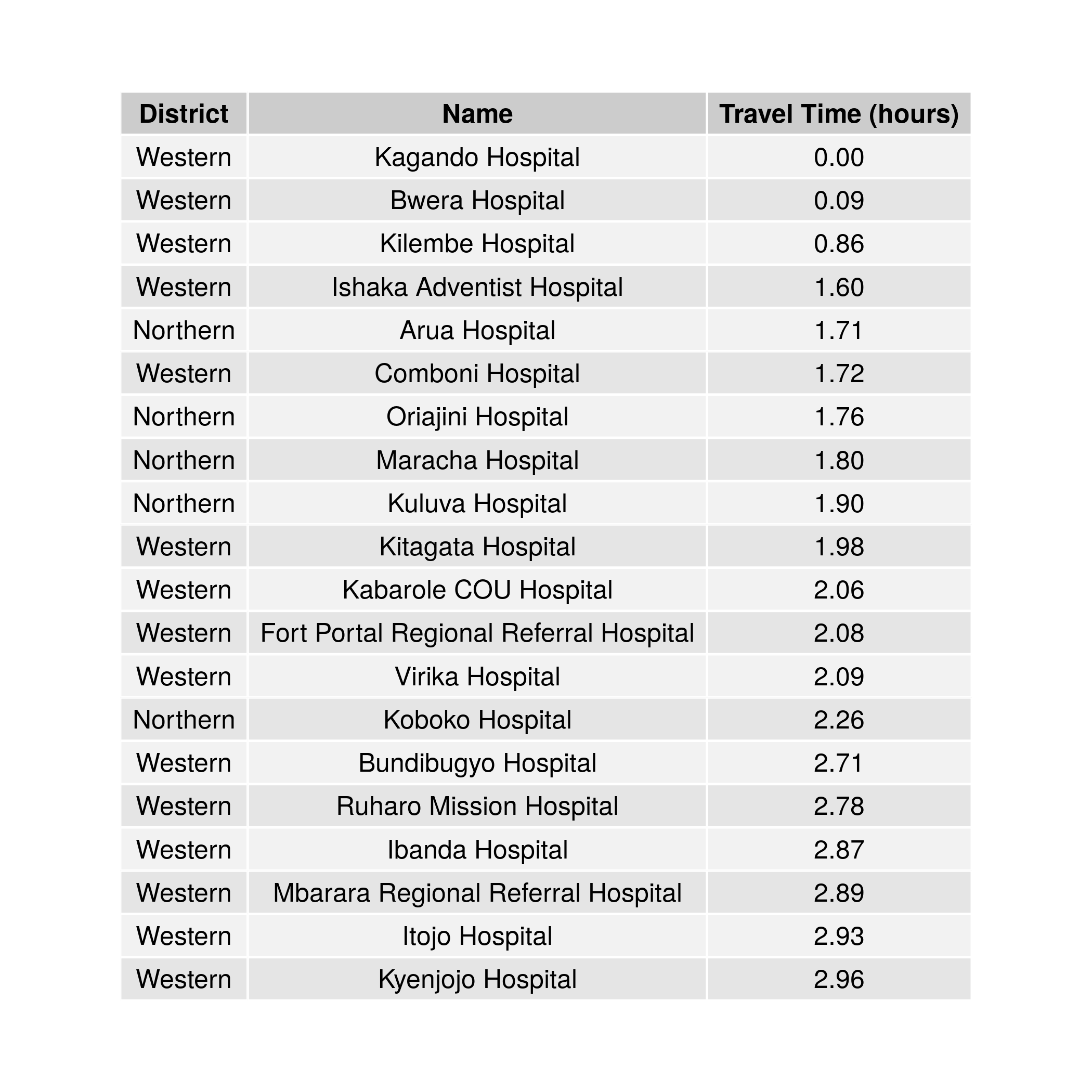


**Table S5. Travel time to most accessible hospital from locations with Ebola cases (2018-2019), Rwanda.** This table presents the travel times from the twenty closest hospitals in Rwanda to a location with Ebola cases based on case data from the WHO’s Situation Report 50, published July 16, 2019. The hospitals are presented in rank-order from closest (shortest travel times) to furthest (longest travel time) among the twenty.


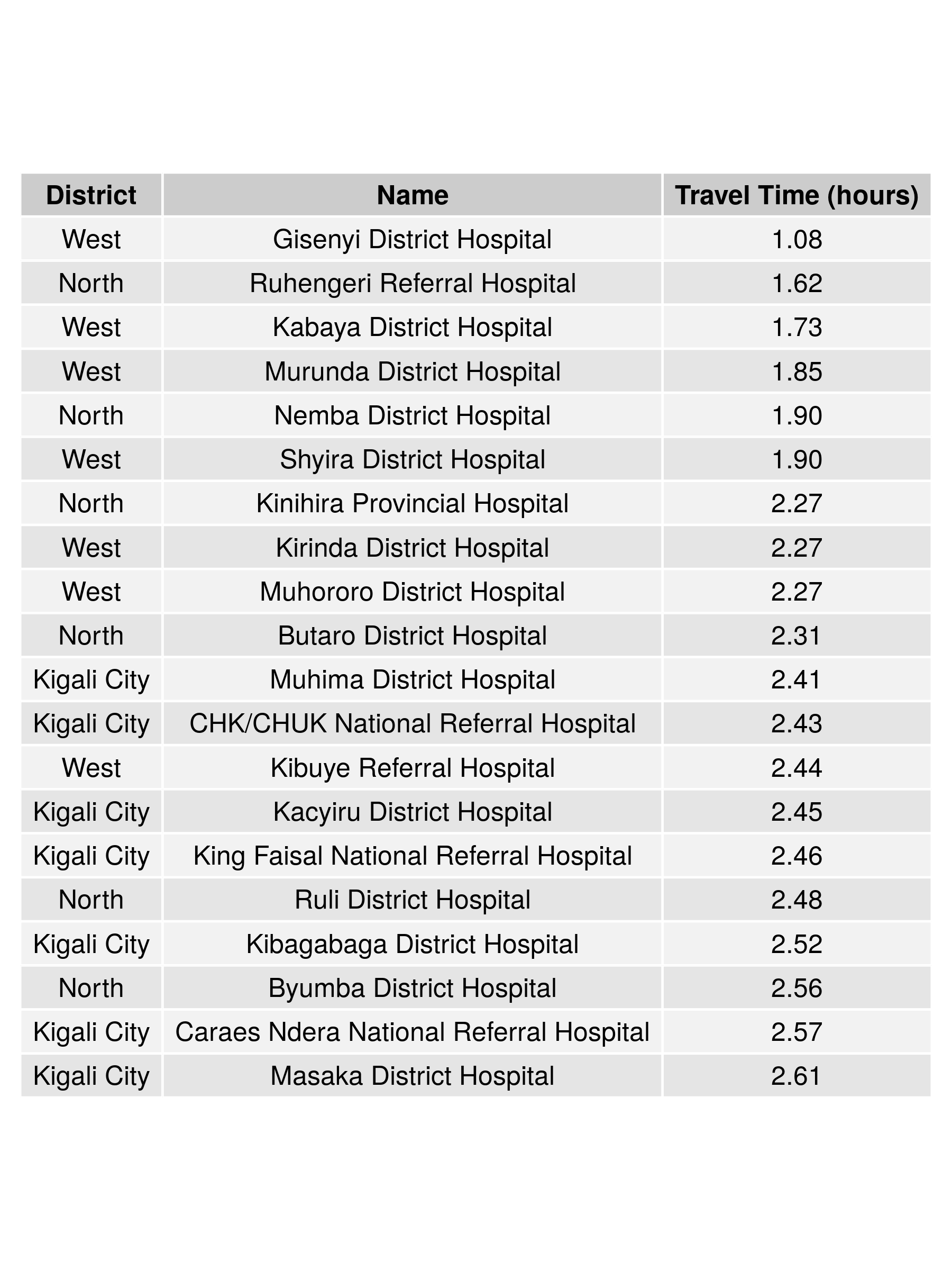


**Table S6 Travel time to most accessible hospital from locations with Ebola cases (2018-2019), South Sudan.** This table presents the travel times from the twenty closest hospitals in South Sudan to a location with Ebola cases based on case data from the WHO’s Situation Report 49, published July 9, 2019. The hospitals are presented in rank-order from closest (shortest travel times) to furthest (longest travel time) among the twenty.


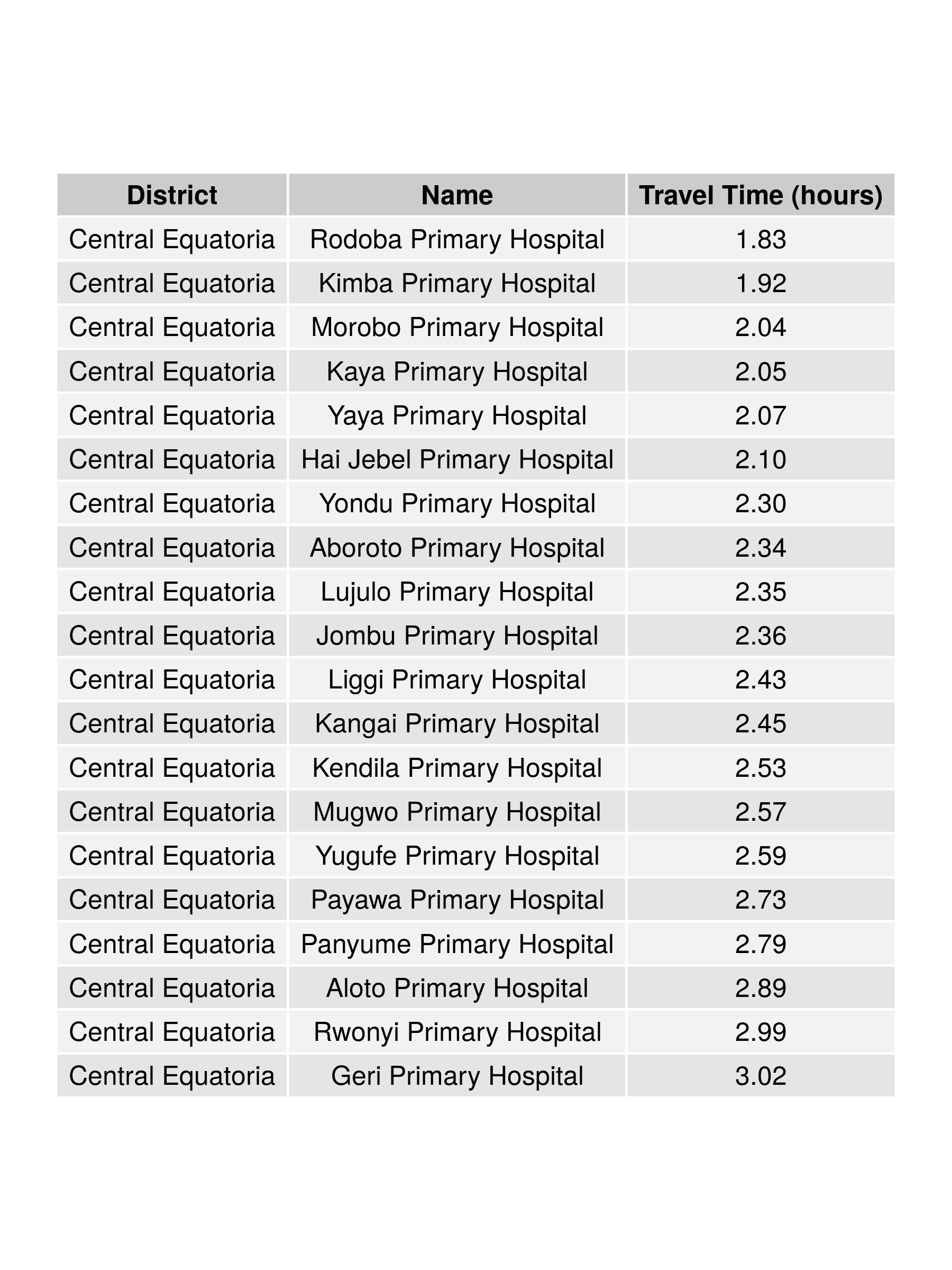


**Table S7 Terms used for facility stratification by country.** This table presents the terms used for facility type stratification and the original terminology recoded.

| **Country** | **Facility Type (Original)** | **Facility Type (Recoded)** |
| --- | --- | --- |
| Angola | Hospital | Hospital |
| Angola | Municipal Hospital | Hospital |
| Angola | Provincial Hospital | Hospital |
| Angola | Posto de Saúde | Health Post |
| Angola | Centro de Saúde | Health Centre |
| Angola | Centro Materno Infantil | Maternity |
| Angola | Central Hospital | Hospital |
| Angola | Centro Sanatorio Materno Infantil | Maternity |
| Angola | Regional Hospital | Hospital |
| Angola | General Hospital | Hospital |
| Benin | Health Centre | Health Centre |
| Benin | Community Health Centre | Community Health Unit |
| Benin | Hôpital de Zone | Hospital |
| Benin | Centre de Santé d’Arrondissement | Health Centre |
| Benin | Dispensaire | Dispensary |
| Benin | Centre de Santé de Sous-Prefecture | Health Centre |
| Benin | Centre Médical | Medical Center |
| Benin | Unites de Santé de Village | Community Health Unit |
| Benin | Centre Hospitalier Départemental | Hospital |
| Benin | Centro de Santé de Circonscription Urbaine | Health Centre |
| Benin | Hôpital | Hospital |
| Benin | Clinic | Health Clinic |
| Benin | Centre de Santé Central | Health Centre |
| Benin | Centre National Hospitalier Universitaire | Hospital |
| Benin | Centre Médico-social | Medical Center |
| Botswana | Clinic | Health Clinic |
| Botswana | Primary Hospital | Health Clinic |
| Botswana | Health Post | Health Post |
| Botswana | District Hospital | Hospital |
| Botswana | Referral Hospital | Hospital |
| Burkina Faso | Centre de Santé et de Promotion Sociale | Health Centre |
| Burkina Faso | Centre Médical Avec Antenne Chirurgicale | Medical Center |
| Burkina Faso | Centre Hospitalier Régional | Hospital |
| Burkina Faso | Centre Médical | Medical Center |
| Burkina Faso | Dispensaire | Dispensary |
| Burkina Faso | Centre Hospitalier National | Hospital |
| Burkina Faso | Centre Hospitalier Universitaire National | Hospital |
| Burundi | Hôpital de District | Hospital |
| Burundi | Health Centre | Health Centre |
| Burundi | Hôpital Tertiaire | Hospital |
| Cameroon | Health Centre | Health Centre |
| Cameroon | Centre de Santé Intégré | Health Centre |
| Cameroon | Hôpital de District | Hospital |
| Cameroon | Centre Medical d’Arrondissement | Medical Center |
| Cameroon | Hôpital Régional | Hospital |
| Cameroon | Dispensaire | Dispensary |
| Cameroon | Hôpital Centraux | Hospital |
| Cameroon | Hôpital Général | Hospital |
| Cameroon | Clinic | Health Clinic |
| Central African Republic | Centre de Santé ""C"" | Health Centre |
| Central African Republic | Hôpital Centraux | Hospital |
| Central African Republic | Centre de Santé ""E"" | Health Centre |
| Central African Republic | Centre de Santé ""B"" | Health Centre |
| Central African Republic | Centre de Santé ""A"" | Health Centre |
| Central African Republic | Poste de santé | Health Post |
| Central African Republic | Centre de Sante | Health Centre |
| Central African Republic | Hôpital Régional Universitaire | Hospital |
| Central African Republic | Hôpital Préfectoraux | Hospital |
| Central African Republic | Centre de Santé ""D"" | Health Centre |
| Chad | Health Centre | Health Centre |
| Chad | Hôpital de District | Hospital |
| Chad | Hôpital de Regional | Hospital |
| Chad | Hôpital de Nationaux | Hospital |
| Chad | Regional hospital | Hospital |
| Congo | Centre de Santé Intégré | Health Centre |
| Congo | Hôpital Comboutique | Hospital |
| Congo | l’Hôpital de Base | Hospital |
| Congo | University Hospital | Hospital |
| Congo | Hôpital Général | Hospital |
| Congo | Hôpital | Hospital |
| Cote d'Ivoire | Centre de Santé Rural | Health Centre |
| Cote d'Ivoire | Centre de Santé Urbain | Health Centre |
| Cote d'Ivoire | Centre Médico-social | Medical Center |
| Cote d'Ivoire | Hôpital Général | Hospital |
| Cote d'Ivoire | Hospitalier Universitaire | Hospital |
| Cote d'Ivoire | Hospitalier Régional | Hospital |
| Democratic Republic of the Congo | Poste de Santé | Health Post |
| Democratic Republic of the Congo | Centre de Santé | Health Centre |
| Democratic Republic of the Congo | Hôpital Général de Référence | Hospital |
| Democratic Republic of the Congo | Dispensaire | Dispensary |
| Democratic Republic of the Congo | Centre Médical | Medical Center |
| Democratic Republic of the Congo | Centre Hôpital | Hospital |
| Democratic Republic of the Congo | Centre de Santé de Référence | Health Centre |
| Democratic Republic of the Congo | Polyclinique | Polyclinic |
| Democratic Republic of the Congo | Clinique | Health Clinic |
| Democratic Republic of the Congo | Centre de Santé Municipal | Health Centre |
| Democratic Republic of the Congo | Hôpital | Hospital |
| Democratic Republic of the Congo | Centre Medico-Chirurgical | Medical Center |
| Djibouti | Health Post | Health Post |
| Djibouti | Hospital Medical Center | Hospital |
| Djibouti | Hospital Medical Centre | Hospital |
| Djibouti | Tertiary Hospital | Hospital |
| Djibouti | Community Health Centre | Community Health Unit |
| Equatorial Guinea | Regional Hospital | Hospital |
| Equatorial Guinea | Health Centre | Health Centre |
| Equatorial Guinea | District Hospital | Hospital |
| Eritrea | Health Centre | Health Centre |
| Eritrea | Health Station | Health Station |
| Eritrea | Clinic | Health Clinic |
| Eritrea | Hospital | Hospital |
| Eritrea | Mini Hospital | Hospital |
| Eritrea | Mini Clinic | Health Clinic |
| Eritrea | National Referral Hospital | Hospital |
| Ethiopia | Clinic | Health Clinic |
| Ethiopia | Health Centre | Health Centre |
| Ethiopia | Health Post | Health Post |
| Ethiopia | Hospital | Hospital |
| Ethiopia | Health Station | Health Station |
| Ethiopia | Nucleas Health Centre | Health Centre |
| Ethiopia | National Hospital | Hospital |
| Ethiopia | General Hospital | Hospital |
| Ethiopia | District Hospital | Hospital |
| Ethiopia | Zonal Hospital | Hospital |
| Ethiopia | Referral Hospital | Hospital |
| Gabon | Dispensaire | Dispensary |
| Gabon | Centre de Santé Urbain | Health Centre |
| Gabon | Health Centre | Health Centre |
| Gabon | Regional Hospital | Hospital |
| Gabon | Medical Centre | Medical Center |
| Gabon | Centre Hospitalier Urbain | Hospital |
| Gabon | University Hospital | Hospital |
| Gabon | Hôpital Coopération | Hospital |
| Gambia | Hospital | Hospital |
| Gambia | Health Centre (minor) | Health Centre |
| Gambia | Clinic | Health Clinic |
| Gambia | Health Centre (major) | Health Centre |
| Gambia | Teaching Hospital | Hospital |
| Gambia | General Hospital Hospital | Hospital |
| Ghana | Clinic | Health Clinic |
| Ghana | Health Centre | Health Centre |
| Ghana | Community-based Health Planning and Services | Community Health Unit |
| Ghana | Hospital | Hospital |
| Ghana | District Hospital | Hospital |
| Ghana | General Hospital | Hospital |
| Ghana | Teaching Hospital | Hospital |
| Ghana | Regional Hospital | Hospital |
| Ghana | Municipal Hospital | Hospital |
| Ghana | Polyclinic | Polyclinic |
| Guinea | Poste de Santé | Health Post |
| Guinea | Health Centre | Health Centre |
| Guinea | Hôpital Régional | Hospital |
| Guinea | Hôpital Prefectoral | Hospital |
| Guinea | National Hospital | Hospital |
| Guinea Bissau | Regional Hospital | Hospital |
| Guinea Bissau | Hospital | Hospital |
| Guinea Bissau | National Hospital | Hospital |
| Kenya | Dispensary | Dispensary |
| Kenya | Health Centre | Health Centre |
| Kenya | District Hospital | Hospital |
| Kenya | Sub-District Hospital | Hospital |
| Kenya | Mission Hospital | Hospital |
| Kenya | Clinic | Health Clinic |
| Kenya | Hospital | Hospital |
| Kenya | County Referral Hospital | Hospital |
| Kenya | Provincial General Hospital | Hospital |
| Kenya | National Referral Hospital | Hospital |
| Lesotho | District Hospital | Hospital |
| Lesotho | Health Centre | Health Centre |
| Lesotho | Filter Clinic | Health Clinic |
| Lesotho | Mission Hospital | Hospital |
| Lesotho | National Referral Centre | Hospital |
| Liberia | Clinic | Health Clinic |
| Liberia | Hospital | Hospital |
| Liberia | Health Centre | Health Centre |
| Liberia | Mission Hospital | Hospital |
| Liberia | National Referral Hospital | Hospital |
| Madagascar | Hospital | Hospital |
| Madagascar | Health Centre | Health Centre |
| Madagascar | Health Post | Health Post |
| Malawi | Clinic | Health Clinic |
| Malawi | Health Centre | Health Centre |
| Malawi | Community Hospital | Community Health Unit |
| Malawi | Health Post/Dispensary | Health Post |
| Malawi | District Hospital | Hospital |
| Malawi | Mission Hospital | Hospital |
| Malawi | Rural Hospital | Hospital |
| Malawi | Central Hospital | Hospital |
| Mali | Clinic | Health Clinic |
| Mali | Community Health Centre | Community Health Unit |
| Mali | Polyclinic | Polyclinic |
| Mali | Hospital | Hospital |
| Mali | University Hospital | Hospital |
| Mali | Referral Health Centre | Hospital |
| Mali | Regional Hospital | Hospital |
| Mauritania | Health Centre | Health Centre |
| Mauritania | Hospital | Hospital |
| Mauritania | Health Post | Health Post |
| Mauritania | General Hospital | Hospital |
| Mozambique | Centro de Saúde Rural I | Health Centre |
| Mozambique | Posto de Saúde | Health Post |
| Mozambique | Centro de Saúde Rural II | Health Centre |
| Mozambique | Centro de Saúde Urbano C | Health Centre |
| Mozambique | Hospital Rural | Hospital |
| Mozambique | Centro de Saúde Urbano B | Health Centre |
| Mozambique | Hospital Provincial | Hospital |
| Mozambique | Hospital Distrital | Hospital |
| Mozambique | Centro de Saúde Urbano A | Health Centre |
| Mozambique | Hospital Geral | Hospital |
| Mozambique | Hospital Central | Hospital |
| Namibia | Clinic | Health Clinic |
| Namibia | Health Centre | Health Centre |
| Namibia | District Hospital | Hospital |
| Namibia | Mission Hospital | Hospital |
| Namibia | Intermediate Hospital | Hospital |
| Namibia | Central Hospital | Hospital |
| Niger | Integrated Health Centre | Health Centre |
| Niger | Health Hut | Health Hut |
| Niger | Hospital | Hospital |
| Niger | Centre Hospitalier Universitaire | Hospital |
| Nigeria | Primary Health Centre | Health Clinic |
| Nigeria | Health Centre | Health Centre |
| Nigeria | Clinic | Health Clinic |
| Nigeria | Health Post | Health Post |
| Nigeria | Model Health Centre | Health Centre |
| Nigeria | General Hospital | Hospital |
| Nigeria | Dispensary | Dispensary |
| Nigeria | Basic Health Centre | Health Centre |
| Nigeria | Comprehensive Health Centre | Health Centre |
| Nigeria | Federal Medical Centre | Medical Center |
| Nigeria | Cottage Hospital | Hospital |
| Nigeria | Polyclinic | Polyclinic |
| Nigeria | Medical Centre | Medical Center |
| Nigeria | Hospital | Hospital |
| Nigeria | University Teaching Hospital | Hospital |
| Nigeria | Model Primary Health Centre | Health Clinic |
| Nigeria | Rural Hospital | Hospital |
| Nigeria | District Hospital | Hospital |
| Nigeria | Natonal Hospital | Hospital |
| Nigeria | State Hospital | Hospital |
| Nigeria | DISPENSARY | Dispensary |
| Rwanda | Health Centre | Health Centre |
| Rwanda | Health Post | Health Post |
| Rwanda | District Hospital | Hospital |
| Rwanda | Referral Hospital | Hospital |
| Rwanda | Provincial Hospital | Hospital |
| Rwanda | National Referral Hospital | Hospital |
| Rwanda | Secondary Health Post | Health Post |
| Senegal | Centre Hospitalier Universitaire | Hospital |
| Senegal | Poste de Santé | Health Post |
| Senegal | Centre Hospitalier National | Hospital |
| Senegal | Health Centre | Health Centre |
| Senegal | Hôpital | Hospital |
| Senegal | Hôpital Général | Hospital |
| Senegal | Hôpital Régional | Hospital |
| Sierra Leone | Mission Hospital | Hospital |
| Sierra Leone | Community Health Centre | Community Health Unit |
| Sierra Leone | Community Health Post | Community Health Unit |
| Sierra Leone | Maternal & Child Health Post | Health Post |
| Sierra Leone | Hospital | Hospital |
| Sierra Leone | Clinic | Health Clinic |
| Sierra Leone | Health Centre | Health Centre |
| Sierra Leone | Health Post | Health Post |
| Sierra Leone | Refferal Hospital | Hospital |
| Somalia | Health Post | Health Post |
| Somalia | Maternal & Child Health Centre | Health Centre |
| Somalia | Regional Hospital | Hospital |
| Somalia | Hospital | Hospital |
| Somalia | Health Centre | Health Centre |
| Somalia | District Hospital | Hospital |
| Somalia | Referral Hospital | Hospital |
| South Africa | District Hospital | Hospital |
| South Africa | Satellite Clinic | Health Clinic |
| South Africa | Clinic | Health Clinic |
| South Africa | Community Health Centre | Community Health Unit |
| South Africa | Regional Hospital | Hospital |
| South Africa | Health Post | Health Post |
| South Africa | Provincial Tertiary Hospital | Hospital |
| South Africa | National Central Hospital | Hospital |
| South Africa | Medical Centre | Medical Center |
| South Africa | Community Health Centre/Clinic | Community Health Unit |
| South Africa | Community Health Centre (After hours) | Community Health Unit |
| South Sudan | Primary Health Care Unit | Health Clinic |
| South Sudan | Primary Health Care Centre | Health Clinic |
| South Sudan | State Hospital | Hospital |
| South Sudan | Teaching Hospital | Hospital |
| South Sudan | County Hospital | Hospital |
| Sudan | Type D Hospital | Hospital |
| Sudan | Hospital | Hospital |
| Sudan | Type C Hospital | Hospital |
| Sudan | Type B Hospital | Hospital |
| Sudan | Type A Hospital | Hospital |
| Sudan | Teaching Hospital | Hospital |
| Sudan | Referral Hospital | Hospital |
| Sudan | National Hospital | Hospital |
| Tanzania | Health Centre | Health Centre |
| Tanzania | Dispensary | Dispensary |
| Tanzania | Hospital | Hospital |
| Tanzania | Referral Hospital | Hospital |
| Tanzania | Designated District Hospital | Hospital |
| Tanzania | District Hospital | Hospital |
| Tanzania | Regional Referral Hospital | Hospital |
| Tanzania | National Hospital | Hospital |
| Togo | Centre Médico-social | Medical Center |
| Togo | Unité de Soins Périphérique | Community Health Unit |
| Togo | Centre Hospitalier Préfectoral | Hospital |
| Togo | Centre Hospitalier Régional | Hospital |
| Togo | Centre Hospitalier Universitaire | Hospital |
| Uganda | Health Centre III | Health Centre |
| Uganda | Clinic | Health Clinic |
| Uganda | Health Centre II | Health Centre |
| Uganda | Health Centre IV | Health Centre |
| Uganda | Hospital | Hospital |
| Uganda | National Referral Hospital | Hospital |
| Uganda | Regional Referral Hospital | Hospital |
| Zambia | Health Centre | Health Centre |
| Zambia | Health Post | Health Post |
| Zambia | Level 1 Hospital | Hospital |
| Zambia | Level 2 Hospital | Hospital |
| Zambia | Rural Health Centre | Health Centre |
| Zambia | Clinic | Health Clinic |
| Zambia | Level 3 Hospital | Hospital |
| Zimbabwe | Clinic | Health Clinic |
| Zimbabwe | Central Hospital | Hospital |
| Zimbabwe | Rural Hospital | Hospital |
| Zimbabwe | Rural Health Clinic | Health Clinic |
| Zimbabwe | District Hospital | Hospital |
| Zimbabwe | Provincial Hospital | Hospital |
| Zimbabwe | District/Provincial Hospital | Hospital |
